# Resistance evolution can disrupt antibiotic exposure protection through competitive exclusion of the protective species

**DOI:** 10.1038/s41396-022-01285-w

**Published:** 2022-07-20

**Authors:** Angus M. Quinn, Michael J. Bottery, Harry Thompson, Ville-Petri Friman

**Affiliations:** 1 School of Life Sciences, University of Warwick, Coventry, UK; 2 Department of Biology, University of York, York, UK; 3 Division of Evolution, Infection and Genomics, School of Biological Sciences, University of Manchester, Manchester, UK

**Keywords:** Microbial ecology, Microbial communities, Antibiotics, Bacterial genetics, Evolution

## Abstract

Antibiotic degrading bacteria can reduce the efficacy of drug treatments by providing antibiotic exposure protection to pathogens. While this has been demonstrated at the ecological timescale, it is unclear how exposure protection might alter and be affected by pathogen antibiotic resistance evolution. Here, we utilised a two-species model cystic fibrosis (CF) community where we evolved the bacterial pathogen *Pseudomonas aeruginosa* in a range of imipenem concentrations in the absence or presence of *Stenotrophomonas maltophilia*, which can detoxify the environment by hydrolysing β-lactam antibiotics. We found that *P. aeruginosa* quickly evolved resistance to imipenem via parallel loss of function mutations in the *oprD* porin gene. While the level of resistance did not differ between mono- and co-culture treatments, the presence of *S. maltophilia* increased the rate of imipenem resistance evolution in the four μg/ml imipenem concentration. Unexpectedly, imipenem resistance evolution coincided with the extinction of *S. maltophilia* due to increased production of pyocyanin, which was cytotoxic to *S. maltophilia*. Together, our results show that pathogen resistance evolution can disrupt antibiotic exposure protection due to competitive exclusion of the protective species. Such eco-evolutionary feedbacks may help explain changes in the relative abundance of bacterial species within CF communities despite intrinsic resistance to anti-pseudomonal drugs.

## Introduction

The evolution of antibiotic resistance is one of the most serious challenges facing modern healthcare [1], causing over 1.2 million deaths and tens of millions of extra days in recovery per year [2, 3]. While the evolution of antibiotic resistance has been extensively studied in laboratory monocultures [4], we still have limited understanding of how antibiotic resistance evolves in more complex multi-species communities, particularly during polymicrobial infections [5, 6]. Several studies have shown that the presence of non-target microbial species can increase or decrease the effectiveness of antibiotic treatments [7–18]. For example, staphylococcal protein-A produced by *Staphylococcus aureus* can interact with the psl-polysaccharide produced by *Pseudomonas aeruginosa*, altering biofilm architecture and increasing the tobramycin resistance of both pathogen species [9]. Similarly, the presence of antibiotic-resistant *Stenotrophomonas maltophilia* has been shown to provide antibiotic exposure protection to *P. aeruginosa* by hydrolysing imipenem and detoxifying the environment [10]. While these studies demonstrate that ecological interactions are likely to alter the strength of antibiotic treatments, the effect inter-species interactions have on the rate and trajectory of de novo resistance evolution is not yet well understood [19–24].

Cystic fibrosis (CF) is a recessive genetic disease that results in the dysfunction of the CF transmembrane regulator ion channel, leading to improper ion homoeostasis and the overproduction of thick mucus which impairs mucociliary clearance [25]. As a result, people with CF show reduced mucosal immunity and are highly prone to chronic bacterial lung infections [26]. While bacterial CF lung communities are often polymicrobial, *P. aeruginosa* is the main pathogen responsible for bacterial-associated pneumonia [27, 28], chronically infecting up to 80% of CF patients [29–32]. Other common CF-associated bacterial species include *S. aureus*, *Burkholderia cepacia*, *Haemophilus influenzae* and *S. maltophilia* [33–35]. Of these, *S. maltophilia* is an emerging respiratory pathogen that persistently infects roughly 10–15% of CF patients [32, 36–39] and is increasingly associated with the presence of *P. aeruginosa*, particularly in older patients [38, 40]. One reason for this could be that *S. maltophilia* possesses a broad spectrum of resistance against many antibiotics commonly used to treat *P. aeruginosa*, most notably carbapenems [41, 42], which might enable *S. maltophilia* to persist in the lung when other pathogenic species have been eradicated through antibiotic treatments [5].

The high carbapenem resistance of *S. maltophilia* is primarily caused by the expression of two chromosomally encoded extracellular β-lactamases, *bla*_L1_ and *bla*_L2_ [43–46], that hydrolyse antibiotics and detoxify the environment. This resistance mechanism could also promote the persistence of sensitive lung bacterial species, and clinical *S. maltophilia* isolates have been shown to protect *P. aeruginosa* from the carbapenem imipenem in laboratory conditions [10, 47]. Specifically, β-lactamase mediated hydrolysis of imipenem by *S. maltophilia* was shown to provide antibiotic exposure protection to the sensitive *P. aeruginosa* PA01 strain in co-cultures, increasing the minimum inhibitory concentration (MIC) of *P. aeruginosa* by up to 16-fold [10]. As expected, this effect was stronger when the relative density of *S. maltophilia* was higher and the concentration of antibiotics low. While no *de novo* resistance evolution was observed during these relatively short-term experiments [10], it remains unclear how exposure protection might alter and be affected by *P. aeruginosa* antibiotic resistance evolution over a longer timescale.

*P. aeruginosa* rapidly evolves in the complex CF lung environment over the course of chronic infection [48]. In addition to antibiotic treatments, these adaptations are driven by multiple factors, including lung environment, host immunity and the presence of competing bacterial species [48, 49]. How these ecological factors interact in determining the trajectory of *P. aeruginosa* antibiotic resistance evolution remains unclear. Here, we specifically focused on investigating the effect of antibiotic exposure protection by *S. maltophilia* on the resistance evolution of sensitive *P. aeruginosa*. Exposure protection could shape the trajectory of antibiotic resistance evolution in several ways. First, it could potentially weaken the selection for resistance by reducing the selective concentration of antibiotics in co-cultures, improving the survival of *P. aeruginosa* and reinforcing the ecological interaction between both species. Reduction in the level of antibiotics could further alter the selection for specific resistance mutations, as sub-MIC concentrations have been shown to change the trajectory of molecular resistance evolution in *Salmonella enterica* by driving the sequential accumulation of small-effect epistatic resistance mutations [50]. Second, it is also possible that exposure protection by *S. maltophilia* could facilitate *P. aeruginosa* resistance evolution by supporting faster growth and increasing the supply rate of resistance mutations [51]. Third, resistance evolution by the sensitive species is likely to reduce the importance of exposure protection, leading to potential eco-evolutionary feedbacks where the initial ecological interaction drives, and is changed as a result of, resistance evolution [20, 52].

To study these questions, we conducted a 24-day long in vitro evolution experiment where we mimicked the nutritional and structural complexity of CF lung sputum using synthetic cystic fibrosis medium (SCFM) supplemented with porcine mucin [53]. This system allowed the continued coexistence of *P. aeruginosa* PA01 and clinical *S. maltophilia* strains in the absence of antibiotics alongside systematic testing of *P. aeruginosa* resistance evolution at clinically relevant β-lactam imipenem concentrations in the absence and presence of *S. maltophilia*. Changes in *P. aeruginosa* population density and antibiotic resistance evolution were tracked temporally during the experiment and underlying resistance mutations determined by sequencing. We found that the presence of *S. maltophilia* led to an equal, and in one treatment increased, rate of imipenem resistance evolution via parallel loss of function mutations in the oprD porin gene. Unexpectedly, resistant *P. aeruginosa* drove *S. maltophilia* into extinction due to increased production of pyocyanin in the presence of imipenem, which was cytotoxic to *S. maltophilia*. Together, these results suggest that the effect of antibiotic exposure protection can be disrupted by chromosomal resistance evolution in the susceptible species, leading to eco-evolutionary feedback through competitive exclusion of the protective species.

## Results

### Clinical *S. maltophilia* strain protects sensitive *P. aeruginosa* PA01 from imipenem

**Fig. 1:**
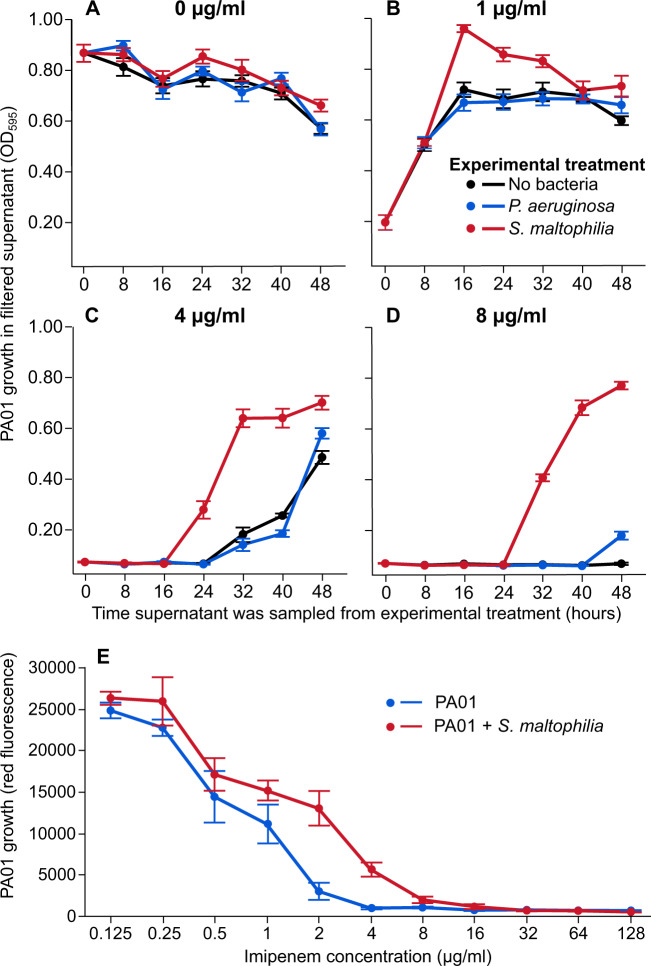
*S. maltophilia* can detoxify imipenem in culture media promoting *P. aeruginosa* growth. The ability of *S. maltophilia* to degrade imipenem was measured indirectly in SCFM (**A**–**D**; *N* = 6). SFCM with 0 µg/ml (A), 1 µg/ml (**B**), 4 µg/ml (**C**) and 8 µg/ml (**D**) imipenem was inoculated with either ancestral *P. aeruginosa* PA01 (blue), *S. maltophilia* (red) or no bacteria (black) and incubated for 48 h. Cultures were sampled at 8 h intervals and filter sterilised before inoculation with PA01:rfp, which was independently grown in all filtrates for 24 h and growth measured at OD595 to indirectly quantify imipenem degradation. **E** shows MIC curves for PA01:rfp in the presence (red) and absence (blue) of *S. maltophilia* when co-cultured in SCFM (*N* = 12). PA01:rfp-specific growth was measured using red fluorescence intensity: excitation 532 nm, emission 588 nm. All error bars correspond to ±one standard error of the mean.

To ascertain if *S. maltophilia* can provide antibiotic exposure protection to *P. aeruginosa*, we first measured the ability of the *S. maltophilia* CF isolate “518951” to degrade imipenem over time using supernatant assays. SCFM was treated with three imipenem concentrations and inoculated with either PA01 or *S. maltophilia*, alongside “no-inoculum” and “no-antibiotic” treatments. Cultures were sampled every 8 h, filter sterilised, and then re-inoculated with imipenem sensitive PA01, which was grown for 24 h to evaluate changes in the antimicrobial activity of the media. In a previous study, we quantified the chemical degradation of imipenem by the *S. maltophilia* strain “SM-518951” using liquid chromatography-mass spectrometry [10]. The presence of *S. maltophilia* significantly increased the rate of imipenem detoxification, allowing the subsequent growth of *P. aeruginosa* in the filtrate (ANOVA: *F*_1,43_ = 79.60, *p* < 0.001; Fig. 1A–D). This effect was most pronounced in the 4 μg/ml and 8 μg/ml imipenem concentration treatments, where detoxification by *S. maltophilia* improved PA01 growth after 24 h and 32 h, respectively, compared to non-detoxified control treatments (4 μg/ml Welch’s *t* test: MD = 9.32, SED = 0.72, *t*_6.9_ = 12.86, *p* < 0.001; 8 μg/ml: MD = 10.48, SED = 0.32, *t*_5.6_ = 33.27, *p* < 0.001). The presence of *S. maltophilia* had no effect on PA01 growth in the absence of imipenem (Welch’s *t* test: MD = 1.91, SED = 1.66, *t*_9.3_ = 1.15, *p* = 0.28), while improved growth was observed after 16 h of detoxification in the sub-MIC 1 μg/ml concentration (MD = 5.21, SED = 1.34, *t*_8.8_ = 3.90, *p* = 0.0038). Like other carbapenems, imipenem is inherently unstable in aqueous solution [54, 55]. However, natural breakdown of imipenem had only a marginal effect on its antimicrobial activity at above-MIC concentrations during the timescale of the experiment.

The effect of *S. maltophilia* exposure protection on PA01 imipenem sensitivity was further quantified using minimum inhibitory concentration (MIC) assays, where the relative abundance of a fluorescently labelled PA01 isolate “PA01:rfp” was measured in the presence and absence of *S. maltophilia*. The presence of *S. maltophilia* significantly increased PA01 growth in the presence of imipenem (ANOVA: *F*_1,380_ = 10.79, *p* = 0.0011; Fig. 1E), raising the average MIC from 2 μg/ml to 8 μg/ml of imipenem. There was also a significant interaction effect between *S. maltophilia* presence and imipenem concentration (ANOVA: *F*_1,380_ = 4.60, *p* = 0.033), demonstrating the intensity of this protective effect depended on imipenem concentration, which was highest at the 2 μg/ml concentration (Fig. 1E). Overall, these results show that *S. maltophilia* was able to detoxify SCFM, permitting the growth of *P. aeruginosa* PA01 in otherwise inhibitory concentrations of imipenem.

### The presence of *S. maltophilia* increases the rate of *P. aeruginosa* PA01 imipenem resistance evolution

**Fig. 2:**
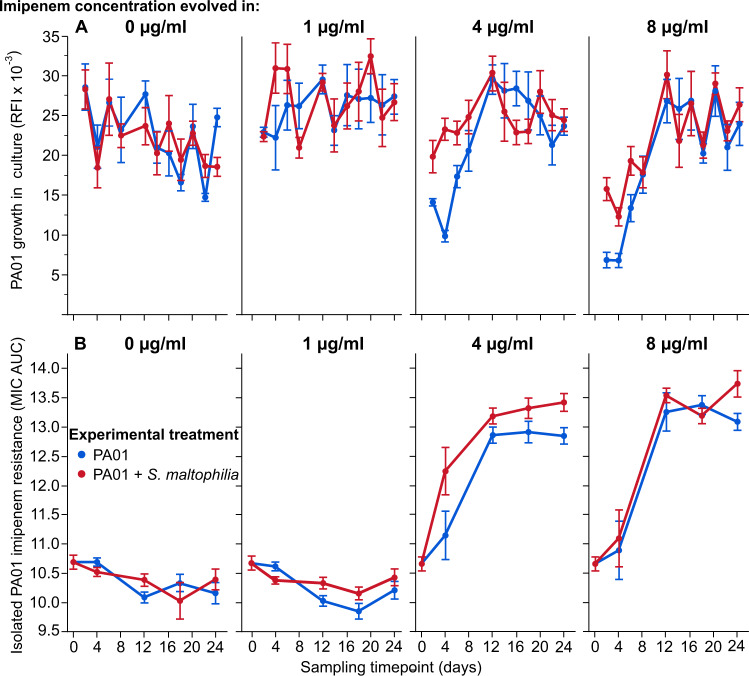
*S. maltophilia* increases *P. aeruginosa* cell densities and promotes the rate of imipenem resistance evolution. The density of *P. aeruginosa* in evolved cultures (**A**) was measured as red fluorescence intensity (RFI) before populations were serially transferred to fresh SCFM treated with either 0 µg/ml, 1 µg/ml, 4 µg/ml or 8 µg/ml imipenem every 2 days. Each sampling point represents the average score across ten independently evolved populations. The evolution of PA01:rfp imipenem resistance was measured by isolating a single *P. aeruginosa* colony from each population and comparing growth across a range of imipenem concentrations using minimum inhibitory concentration (MIC) assays, taking area under the MIC curve (AUC MIC) as a proxy for overall resistance (**B**). Two technical replicates were performed per isolate and error bars correspond to ±one standard error of the mean.

To investigate the consequences exposure protection has on the evolution of imipenem resistance, we evolved the strain PA01:rfp alone and in the presence of *S. maltophilia* in SCFM containing either 0, 1, 4 or 8 μg/ml imipenem for 24 days. We found that compared to monocultures, PA01 reached significantly higher cell densities in the presence of *S. maltophilia* during the first 8 days of the experiment in 4 μg/ml (ANOVA: *F*_1,78_ = 34.56, *p* < 0.001; Fig. 2A) and 8 μg/ml imipenem concentrations (*F*_1,68_ = 13.82, *p* < 0.001; Fig. 2A). The presence of *S. maltophilia* had no effect on *P. aeruginosa* cell density in co-cultures at 0 μg/ml or 1 μg/ml imipenem concentrations (all *p* > 0.05). Moreover, the effect of *S. maltophilia* presence on PA01 growth became non-significant in all imipenem concentrations after 8 days of the experiment.

The imipenem resistance of evolved *P. aeruginosa* isolates from independent populations was compared using area under the MIC curve (MIC AUC) across 11 dilution steps ranging from 0.125 to 128 μg/ml imipenem concentrations (50% dilutions). PA01 evolved resistance to imipenem at both 4 μg/ml (MIC AUC ANOVA: *F*_1,73_ = 47.43, *p* < 0.001) and 8 μg/ml imipenem concentrations (*F*_1,69_ = 31.36, *p* < 0.001), while no resistance was observed at 0 μg/ml (*F*_1,73_ = 3.53, *p* = 0.064) or 1 μg/ml imipenem treatments (*F*_1,73_ = 0.076, *p* = 0.78; Fig. 2B). Imipenem resistance evolution was reflected by an average increase in MIC breakpoints from 1.8 to 18.7 μg/ml across imipenem-resistant PA01 clones. Although the overall level of imipenem resistance did not differ between 4 μg/ml and 8 μg/ml concentration treatments, resistance evolved faster in the presence of *S. maltophilia* in the 4 μg/ml imipenem concentration (as measured by MIC AUC ANOVA: *F*_1,73_ = 11.30, *p* = 0.0012; Fig. 2B, and MIC breakpoint ANOVA: *F*_1,33_ = 6.58, *p* = 0.015; SI Fig. 9). In contrast, the presence of *S. maltophilia* had no significant impact on the rate of resistance at the 8 μg/ml imipenem concentration (ANOVA: *F*_1,69_ = 1.07, *p* = 0.30; Fig. 2B, Individual MIC curves: SI Figs. 1–8). Despite being consistently exposed to above-MIC imipenem concentrations, *P. aeruginosa* went extinct in only one out of 60 replicate populations treated with imipenem (one replicate at 4th day in 8 μg/ml imipenem concentration in the absence of *S. maltophilia*). Together, these results show that the presence of *S. maltophilia* can increase the rate of imipenem resistance evolution in *P. aeruginosa*, without affecting the overall level of resistance.

### Non-synonymous mutations in *oprD* provide resistance to imipenem at a cost to competitive fitness

To investigate the mutations associated with imipenem resistance, we randomly chose one *P. aeruginosa* isolate from six independently evolved replicate populations from each treatment at the end of the selection experiment (day 24). Evolved isolates that had been co-cultured with *S. maltophilia* had an average of one (SD = 1.64) mutation per isolate compared to 0.71 (SD = 0.62) mutations per isolate sequenced from monoculture populations (SI Table 1). Furthermore, *P. aeruginosa* isolates that had evolved in 0 and 1 μg/ml imipenem concentrations had 0.63 (SD = 1.71) mutations per isolate on average, compared to 1.08 (SD = 0.28) mutations per isolate evolved in 4 and 8 μg/ml imipenem concentrations. Most of the observed mutations were non-synonymous (36/41), and of these, 11 were amino acid substitutions, 10 stop codon insertions, 13 frameshifts, one deletion (3332 bp) and one insertion (92 bp). Only mutations in the *oprD* gene were found to evolve parallel across replicates from independently evolved populations.

**Fig. 3:**
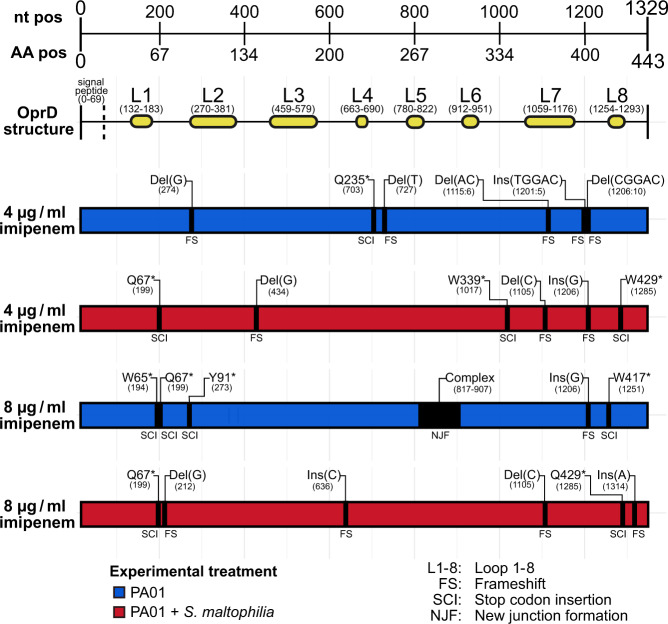
A wide range of non-synonymous mutations in the *oprD* gene explain increased imipenem resistance in evolved *P. aeruginosa*. All imipenem-resistant PA01:rfp isolates had loss of function mutations in the *oprD* gene, which consists of 1329 nucleotides (nt) and encodes a 443 amino acid (AA) product. The OprD peptide contains a small signal peptide followed by eight structural transmembrane loops (L1-8). Each black bar represents a single mutation from a different *P. aeruginosa* isolate evolved in separate populations (Six replicate populations per treatment, 24 replicate populations in total). No imipenem-resistant PA01 isolate possessed more than one mutation in the *oprD* gene. Information on the type of mutations are provided above, denoting for insertions “Ins(nt)”, deletions “Del(nt)” and amino acid (AA) substitutions “original AA—AA position—new AA” (*= stop codon). The nucleotide position in the *oprD* gene for each mutation is shown in brackets. The predicted effect of the mutation on the gene is shown as frameshift (FS) or stop codon insertion (SCI). One isolate had a complex rearrangement of a 91 bp region which led to the formation of a new structural junction (NJF).

We found that all sequenced imipenem-resistant isolates from 4 µg/ml and 8 µg/ml imipenem treatments had non-synonymous mutations in the *oprD* gene encoding the OprD outer membrane porin (24/24 of sequenced isolates; SI Table 1), which has previously been linked with carbapenem resistance in *P. aeruginosa* [56]. Highly parallel mutations at this locus likely represents adaptive evolution. In contrast, none of the isolates from the 0 µg/ml and 1 µg/ml imipenem concentrations had *oprD* mutations, regardless of if they had evolved in the absence or presence of *S. maltophilia*. Parallel *oprD* mutations were found equally often in isolates cultured in the absence and presence of *S. maltophilia* and consisted of a wide variety of insertions, deletions, and single base substitutions across a 1119 bp region within the 1332 bp long gene (Fig. 3). Eight isolates had *oprD* mutations in structural loops (loops: 2, 5, 7 and 8), while the remaining 16 isolates had *oprD* mutations in non-loop regions. All *oprD* mutations resulted in either a change in the reading frame or the introduction of novel stop codons, except for one isolate which possessed a new 91 bp long structural junction (Fig. 3). These mutations thus likely led to deficiency in the function of the outer membrane porin.

**Fig. 4:**
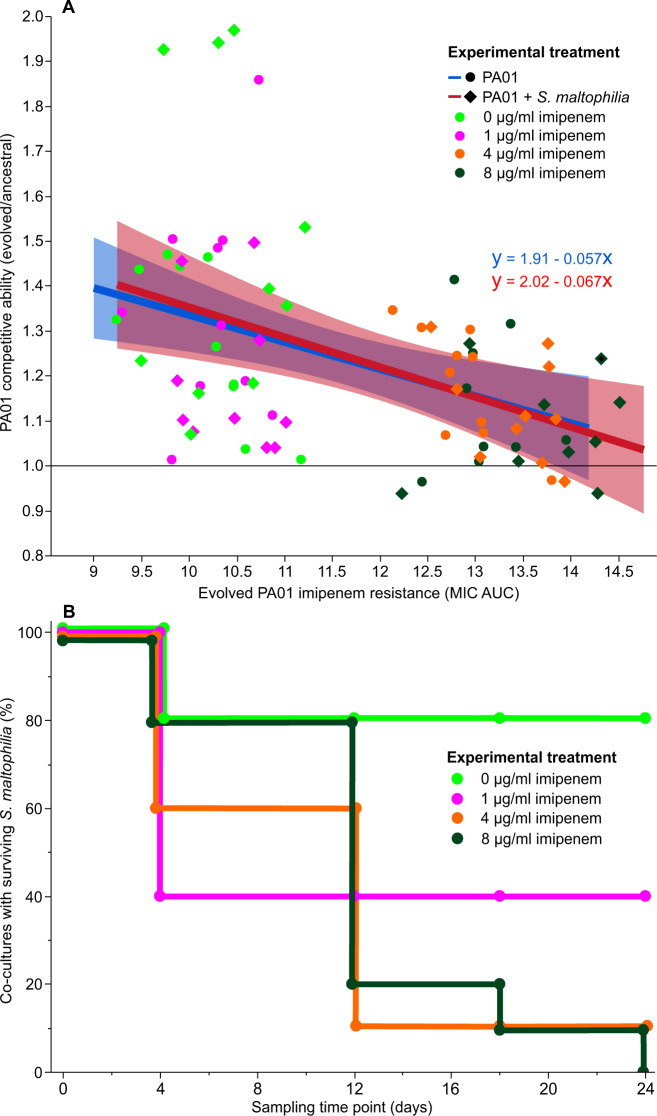
Imipenem resistance decreases *P. aeruginosa* competitive fitness and is associated with *S. maltophilia* extinctions in co-cultures. The evolution of imipenem resistance in *P. aeruginosa* results in a trade-off with competitive fitness (**A**), which was not affected by the absence (blue) or presence (red) of S. maltophilia in evolved cultures (based on the correlation coefficient of the linear regression). Shaded regions represent 95% confidence intervals for fitted regression lines. Imipenem exposure induces the competitive exclusion of resistant *S. maltophilia* by *P. aeruginosa* in co-cultures (**B**). The survival plot shows the percentage of co-culture populations containing *S. maltophilia* (*N* = 10).

To explore if the evolution of imipenem resistance imposed a fitness cost for *P. aeruginosa*, we directly competed evolved PA01:rfp isolates with the non-fluorescent, otherwise isogenic, ancestral PA01 strain in SCFM in the absence of imipenem. Overall, evolved isolates showed higher fitness relative to the ancestral strain (Fig. 4A) after evolving in 0 µg/ml (Dunnett’s test: MD = 0.38, *p* < 0.001), 1 µg/ml, (MD = 0.27, *p* < 0.001) and 4 µg/ml imipenem treated SCFM (MD = 0.16, *p* = 0.011), indicative of media adaptation. No significant difference in fitness was observed between ancestral PA01 and evolved isolates originating from the 8 µg/ml imipenem concentration treatment (MD = 0.12, *p* = 0.059). Changes in the fitness of evolved isolates was not significantly affected by the presence or absence of *S. maltophilia* during the selection experiment (ANOVA: F_1,75_ = 0.050, *p* = 0.82). Across all treatments, we found a negative correlation between evolved isolates’ fitness and imipenem resistance (Multiple Linear Regression: *F*_1,75_ = 18.50, *p* < 0.001, *R*^2^ = 0.21), which was also not affected by previous exposure to *S. maltophilia* (*F*_1,75_ = 0.035, *p* = 0.85). Together, this data suggests that imipenem resistance was costly in terms of constraining *P. aeruginosa* adaptation to the growth media.

Only six out of the 48 sequenced isolates had non-synonymous mutations in non-OprD coding genes. Mutations in these genes did not show any parallelism and were observed only once in individual isolates, which suggests they were unlikely linked with imipenem resistance. Four isolates from the 0 µg/ml imipenem treatment possessed mutations in genes: *mvaT* (S23P), *PA0715* (C201Y and K227E), *PA1874* (G964A, G964C and S1375T), *PA2228:30* (3,332 bp del), *pilA* (S64G) and *pilB* (D388A). One isolate from the 1 µg/ml imipenem treatment had a mutation in the *vfr* gene (H164P) and two isolates evolved in 4 µg/ml imipenem treatment had mutations in PA4041(Y324D) and *parS* (T131P). While most isolates showed improved competitive ability relative to the ancestral strain, four showed strikingly high increases in fitness compared to the other evolved isolates, two of which were included in our sequencing (ANOVA: *F*_1,18_ = 12.35, *p* = 0.0025; *F*_1,15_ = 6.14, *p* = 0.026; Fig. 4A). One high fitness isolate had a single mutation in the *vfr* gene (H164P) while the second had four non-synonymous mutations, one in *pilA* (S64G) and *mvaT* (S23P) genes, alongside two mutations in the *PA0715* gene (C201Y and K227E), indicating that these mutations might have contributed towards the fitness increases observed in SCFM.

To help understand what drove changes in competitive fitness, we compared the ability of evolved isolates and the ancestral PA01 to produce pyoverdine, a siderophore involved in the uptake and sequestration of iron that plays an important role in inter-species resource competition [57, 58]. The majority of evolved PA01 isolates exhibited increased pyoverdine production relative to the ancestral PA01 (Dunnett’s test: *p* < 0.05 for all pairwise comparisons; SI Fig. 10), demonstrating pyoverdine upregulation is likely a key adaptation to growth in SCFM. However, we found no clear difference between isolates evolved in the absence or presence of imipenem (ANOVA: *F*_3,75_ = 1.50, *p* = 0.22) or *S. maltophilia* during the selection experiment (*F*_1,75_ = 0.19, *p* = 0.66; SI Fig. 10), suggesting that competition for iron unlikely explains the trade-off between antibiotic resistance and competitive fitness. In summary, these results demonstrate that imipenem resistance evolved through loss of function mutations in the *oprD* gene that emerged equally often in above-MIC imipenem concentrations (in both 4 and 8 µg/ml) regardless of the presence of *S. maltophilia*, and that the fitness cost of imipenem resistance stemmed from reduced adaptation to the growth media.

### Imipenem resistance evolution coincided with the competitive exclusion of *S. maltophilia* alongside increased production of antimicrobial pyocyanin by *P. aeruginosa*

The survival of *S. maltophilia* was monitored during the selection experiment by determining its presence in co-cultures using selective plating. After 24 days of experimental evolution, *S. maltophilia* could be detected in 80% and 40% of co-cultures in 0 μg/ml and 1 μg/ml imipenem concentration treatments, respectively. In contrast, the survival of *S. maltophilia* decreased over time in above-MIC imipenem concentrations in the presence of *P. aeruginosa* (Fig. 4B; Log-Rank test: *Z* = 10.97, *p* = 0.012) and was driven into extinction in 95% (19 out of 20) of co-culture replicates. Crucially, the disappearance of *S. maltophilia* in 4 μg/ml and 8 μg/ml imipenem treatments coincided with the evolution of increased imipenem resistance in *P. aeruginosa* during the first 12 days of the selection experiment (MIC ANOVA: *F*_1,34_ = 20.82, *p* < 0.001, AUC ANOVA: *F*_1,34_ = 12.88, *p* = 0.0010, Figs. 2B, C,  4B). In contrast to co-cultures, no *S. maltophilia* extinctions were observed in any of the monoculture control replicates regardless of the imipenem concentration. These results suggest that PA01 competitively excluded *S. maltophilia* primarily in above-MIC imipenem concentrations and that *S. maltophilia* extinctions coincided with *P. aeruginosa* imipenem resistance evolution.

**Fig. 5:**
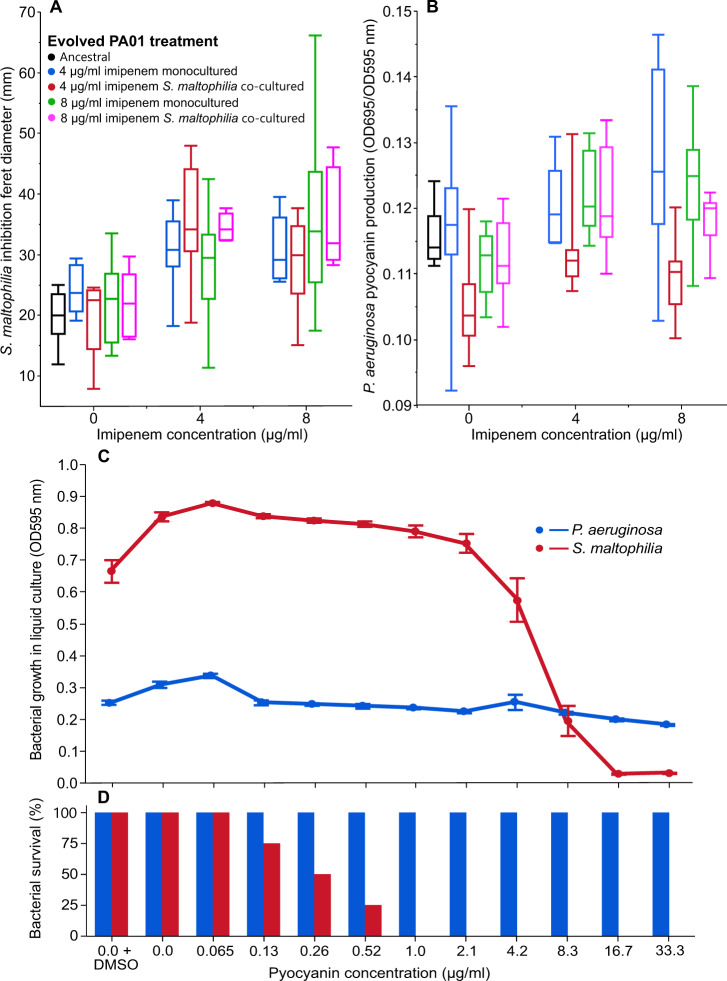
Imipenem exposure increases the inhibition of *S. maltophilia* by *P. aeruginosa* via increased production of cytotoxic pyocyanin. Imipenem treatment increases *S. maltophilia* inhibition by resistant *P. aeruginosa* isolates (**A**) regardless of the evolved antibiotic concentration or the presence of *S. maltophilia* during the selection experiment; Tukey Boxplots represent the interquartile range (25–75th percentile), whiskers show the minimum and maximum values, and the midline shows the median value (*N* = 8). Imipenem treatment increases pyocyanin production in resistant *P. aeruginosa* isolates (**B**). Pyocyanin was measured using optical density (695 nm) and normalised against the bacterial growth in the pre-centrifuged cultures (OD695/OD595) (*N* = 8). Pyocyanin restricts the growth of *S. maltophilia* in liquid culture (**C**), leading to extinctions in concentrations at and above 0.13 µg/ml (**D**). In (**D**), extinctions were determined as no observable growth of replicate populations when spotted on LB agar plates. Confidence intervals represent ±one standard error from the mean (*N* = 4).

To better understand these extinctions, we tested if the presence of imipenem increased *P. aeruginosa*’s antagonism towards *S. maltophilia*. To this end, we cultured imipenem-resistant *P. aeruginosa* isolates in SCFM in the absence and presence of 4 μg/ml or 8 μg/ml of imipenem, before spotting them onto *S. maltophilia* SCFM-agar lawns to compare the level of inhibition. Pre-growing resistant *P. aeruginosa* in the presence of imipenem made isolates significantly more inhibitory against *S. maltophilia* (Mixed model 24 h effect: *F*_1,61_ = 9.98, *p* = 0.0025, *d* = 0.62; 48 h effect: *F*_1,60_ = 23.94, *p* < 0.001, *d* = 1.37; Fig. 5A), regardless of if resistant isolates had evolved in 4 μg/ml or 8 μg/ml imipenem concentrations (Mixed model: *F*_1,28_ = 2.08, *p* = 0.16), or in the presence of *S. maltophilia* during the selection experiment (*F*_1,28_ = 0.057, *p* = 0.81). Variation between isolates accounted for just 7.6% of the total model variation (Wald test: *p* = 0.42). Furthermore, no difference in inhibitory activity against *S. maltophilia* was observed between evolved resistant isolates and the susceptible ancestral PA01:rfp in the absence of imipenem (Dunnett’s test: *p* > 0.05 for all pairwise comparisons), which suggests that this effect was primarily triggered by the presence of antibiotic, instead of prior exposure during the selection experiment.

To explore why resistant *P. aeruginosa* isolates became more inhibitory to *S. maltophilia* in the presence of imipenem, we tested if the presence of imipenem affected the production of cytotoxic pyocyanin, which has previously been shown to be upregulated during antibiotic exposure [59, 60] and to have antimicrobial activity against multiple bacterial species [61, 62]. We observed that culturing resistant *P. aeruginosa* isolates in the presence of imipenem significantly increased the production of pyocyanin (Mixed model: *F*_1,63_ = 21.65, *p* < 0.001, *d* = 0.54; Fig. 5B), while no difference was observed compared to the ancestral PA01:rfp when strains were grown in the absence of imipenem (Dunnett’s tests: *p* > 0.05). These results were unaffected by *P. aeruginosa* isolates that had evolved in either 4 μg/ml or 8 μg/ml imipenem concentrations (*F*_1,29_ = 1.40, *p* = 0.24) during the selection experiment. However, prior presence of *S. maltophilia* led to a marginally lower increase in pyocyanin production (*F*_1,29_ = 9.35, *p* = 0.0048), particularly with clones that had evolved in the 4 μg/ml imipenem treatment. The between-strain variation was relatively large, capturing 35.1% of the total model variation within individual imipenem treatments (Wald test: *p* = 0.023).

Lastly, we experimentally tested the inhibitory effect of *P. aeruginosa* derived pyocyanin on *S. maltophilia* growth using MIC assays. We found that pyocyanin significantly inhibited the growth of *S. maltophilia* (OD595 ANOVA: *F*_1,42_ = 127.05, *p* < 0.001; Fig. 5C) with an MIC of 16.7 μg/ml, while a smaller effect was found with *P. aeruginosa* without a clear MIC point (*F*_1,42_ = 84.50, *p* < 0.001). In line with these results, pyocyanin exposure did not cause any *P. aeruginosa* extinctions at any concentration, while no *S. maltophilia* populations could be revived from cultures treated with above 0.52 μg/ml pyocyanin concentrations (Fig. 5D). Based on the calibration curves of synthetic pyocyanin, we estimate that the average concentrations of pyocyanin produced by imipenem-resistant *P. aeruginosa* were: 3.3 μg/ml pyocyanin (SD = 0.38) in 0 μg/ml imipenem, 4.0 μg/ml pyocyanin (SD = 0.61) in 4 μg/ml imipenem and 4.4 μg/ml pyocyanin (SD = 0.28) in 8 μg/ml imipenem treatments. Together, these results suggest that the evolution of antibiotic resistance in *P. aeruginosa* coincided with the extinction of *S. maltophilia* in co-cultures, likely due to imipenem-induced increase in pyocyanin production.

## Discussion

It is becoming increasingly evident that the ecological context of polymicrobial infection can influence the efficacy of antimicrobial treatments [9, 10, 12, 14, 16, 17]. Here we tested how antibiotic exposure protection, conferred by the hydrolysis of imipenem by co-occurring *S. maltophilia*, might shape the evolution of resistance in the initially susceptible *P. aeruginosa* bacterial pathogen. It was found that while antibiotic exposure protection increased the rate of *P. aeruginosa* resistance evolution in 4 μg/ml imipenem concentrations, it did not lead to significant differences in the overall level of evolved resistance, which were caused by parallel mutations in the outer membrane porin-encoding *oprD* gene. Surprisingly, emergence of imipenem resistance was coupled with the extinction of *S. maltophilia*, while continued coexistence was observed in most *P. aeruginosa* co-cultures in the absence of imipenem. Additional experiments showed that imipenem exposure triggered increased production of pyocyanin by resistant *P. aeruginosa* isolates, which was highly cytotoxic to *S. maltophilia*. Together, these results demonstrate that resistance evolution can disrupt ecologically derived antibiotic exposure protection, driving initially protective species into extinction due to increased interference competition. Resistance evolution could thus drive eco-evolutionary feedbacks in polymicrobial CF communities, partially explaining the rapid species turnover and changes in community composition often observed during in vivo antibiotic treatments.

Resistant community members often provide exposure protection to sensitive species by lowering antibiotic concentrations in the environment [10, 24]. While no difference in the overall level of evolved resistance was found, the presence of *S. maltophilia* increased the rate of de novo imipenem resistance evolution in the 4 μg/ml imipenem concentration. In co-cultures, this was preceded by higher *P. aeruginosa* cell densities which could have improved the supply rate of antibiotic resistance promoting, de novo mutations [51, 63]—an effect that has been observed between pyocyanin producing and pyocyanin deficient *P. aeruginosa* strains during ciprofloxacin treatment [64]. In contrast, exposure protection had no detectable effect on the rate of antibiotic resistance evolution in the 8 μg/ml imipenem concentration. This could be explained by the increased time required for *S. maltophilia* to detoxify the environment at higher imipenem concentrations (Fig. 1C, D), leading to weaker positive effects on *P. aeruginosa* growth. Together, these findings suggests that the presence of *S. maltophilia* leads to an equal rate of *P. aeruginosa* resistance evolution and can, under certain environmental conditions, actively promote resistance evolution compared with monocultured populations. Facilitation of antibiotic resistance evolution by another species is in contrast with previous studies that have found that selection for resistant *E. coli* is reduced in complex bacterial communities [22], or when *E. coli* and *S. enterica* are grown in obligate cross-feeding co-cultures [19]. The presence of other species is often expected to constrain adaptation to the abiotic environment due to costs associated with interspecific resource competition [23, 65, 66]. However, we observed very limited inter-species resource competition in the supernatant and experimental evolution assays, with the presence of *S. maltophilia* having no significant negative effect on *P. aeruginosa*’s growth in the absence of imipenem. While we found that the evolution of imipenem resistance correlated negatively with *P. aeruginosa* competitive fitness, this pattern was primarily driven by the increased fitness of isolates that had evolved in the absence of imipenem, or at sub-MIC concentrations. Selection for prolonged growth in the stationary phase in the absence of imipenem may explain this improved competitive ability as bacterial cells often develop growth advantage at stationary phase (GASP), which is associated with the upregulation of stress-response genes and alternative metabolic pathways [67, 68]. However, as *P. aeruginosa* did not reach stationary phase during 48 h of growth in the presence of 4 or 8 µg/ml imipenem (Fig. 1), antibiotic resistance was selected under exponential growth. As a result, selection at the stationary phase of growth unlikely explained differences in the imipenem resistance or improved fitness of PA01 monocultures. Together, these findings suggest that competition with *S. maltophilia* was relatively weak and that facilitation of resistance evolution via exposure protection could have been driven by positive effects on *P. aeruginosa* growth and a relatively low cost of imipenem resistance.

Exposure protection caused by the removal of antibiotics from the environment could lead to selection for antibiotic resistance taking place in sub-MIC gradients, lowering the strength of selection and changing the trajectory of antibiotic resistance evolution at the molecular level [50]. We found parallel loss of function mutations in the *oprD* gene in all resistant *P. aeruginosa* isolates regardless of whether they had evolved in 4 μg/ml or 8 μg/ml imipenem concentrations in the absence or presence of *S. maltophilia*, which is in line with our phenotypic assays that showed no difference in the overall level of evolved resistance between resistant isolates. These findings closely mirror the molecular basis of imipenem resistance typically observed in clinical strains, which has almost exclusively been linked to deactivation of the *oprD* porin gene [69, 70], involved in the uptake of amino acids, peptides and carbapenem antibiotics [56]. Transmembrane sections of the OprD porin are connected by eight core structural loops [71]. We found that 33% of *oprD* mutants had mutations in either loops 2, 5, 7 and 8, while 67% of the resistant isolates had mutations outside of loop regions. Previous work has shown that targeted deletions in loops 2, 3 and 4 result in decreased imipenem sensitivity and reduced OprD expression [56]. Moreover, polymorphisms in loops 1–7 and frameshift and stop codon insertion mutations in *oprD* have been identified in imipenem-resistant clinical isolates [72–75]. While a variety of mutations were observed in this gene, their locations (e.g. within or outside loops) were not overrepresented in any of our experimental treatments and led to similar levels of imipenem resistance in *P. aeruginosa*. As a result, the evolution of imipenem resistance was not affected by the specific type of mutation in this gene.

Interestingly, we found that the emergence of imipenem resistance was coupled with the extinction of *S. maltophilia* in 4 μg/ml and 8 μg/ml imipenem concentration co-culture treatments. While *P. aeruginosa* is regularly observed to competitively exclude other competitors in vitro [12, 76–79], we observed that both species stably coexisted in the majority of co-cultures in the absence of imipenem, despite *P. aeruginosa* evolving increased competitive fitness. This suggests that the competitive exclusion of *S. maltophilia* was not driven by resource competition and could have instead been caused by the production of secondary metabolites that are often upregulated by *P. aeruginosa* in response to stress via quorum sensing, stringent response or SOS systems [80–83]. We specifically focused on the virulence factor pyocyanin, which is known for its redox activity and cytotoxicity through increased production of environmental H_2_O_2_, depletion of antioxidants and antimicrobial activity against *E. coli*, *S. aureus*, *S. enterica*, *Listeria monocytogenes* and *Bacillus cereus* [84–90]. First, we found that the production of pyocyanin increased in resistant *P. aeruginosa* isolates when exposed to imipenem, regardless of whether they had previously evolved in the presence of *S. maltophilia* or at 4 μg/ml or 8 μg/ml imipenem concentrations. This is in line with previous findings that have shown that *P. aeruginosa* pyocyanin production is often upregulated during antibiotic treatment and can confer a protective effect [60, 64, 78, 91]. Second, pyocyanin was highly cytotoxic to *S. maltophilia*, preventing detectable growth in liquid media at concentrations above 16.7 μg/ml and causing extinctions in concentrations above 0.52 μg/ml. *Stenotrophomonas maltophilia* could thus be relatively more sensitive to pyocyanin compared to other common bacterial CF community members, such as *E. coli*, *S. aureus* and *Kleibsiella* spp., which have pyocyanin MICs of 50, 20 and 40 μg/ml, respectively [61]. Third, pre-growing resistant *P. aeruginosa* strains in the presence of imipenem made them more inhibitory to *S. maltophilia*, when subsequently grown in direct contact on agar plates. Furthermore, the presence of imipenem raised the estimated concentration of pyocyanin production by resistant *P. aeruginosa* to near the threshold required for significant killing of *S. maltophilia* in liquid culture. This is in line with evidence from other studies where *P. aeruginosa* consistently diminishes and inhibits the growth of competitors such as *S. aureus* in co-culture through the production of a mixture of secondary metabolites, including pyocyanin, siderophores and HQNO [34, 92, 93]. Additionally, the formation of metabolites in co-culture by *Streptococcus anginosus* or *Enterobacter aerogenes* can actively stimulate pyocyanin production in *P. aeruginosa* [94, 95]. Together, our findings suggest that the presence of imipenem triggered a phenotypically plastic response in *P. aeruginosa*, leading to increased pyocyanin production and inhibition of *S. maltophilia*. However, production of pyocyanin by *P. aeruginosa* primarily occurs outside of exponential growth in response to nutrient or oxygen limitation and as such is heavily affected by in vitro culture conditions such as shaking, pH and temperature [96, 97]. Even small changes in oxygen availability could alter pyocyanin production, which is particularly relevant within the CF lung where *P. aeruginosa* typically grows along hypoxic gradients that can significantly alter its sensitivity to a range of antibiotics [98, 99]. Although the underlying mechanism of *S. maltophilia* extinction remains unclear, it has previously been demonstrated that mutations in the *oprD* gene can lead to transcriptional changes in numerous *P. aeruginosa* genes, including the upregulation of *phzA1* [100]. This gene governs the production of phenazine-1-carboxylic acid (PCA), which is further converted into pyocyanin by two modifying enzymes PhzM and PhzS [101]. Although we did not observe increased pyocyanin production by *oprD*-mutants in the absence of imipenem, OprD deactivation may have supported increased pyocyanin upregulation during antibiotic treatment. Phenotypically, *oprD* mutants have also been shown to be more resistant to killing by acidic pH, normal human serum and to have increased cytotoxicity against murine macrophages [100]. Our results further show that imipenem resistance evolution can trigger the exclusion of a co-occurring CF pathogen and that the *oprD* gene is also linked to inter-species competition.

The extinction of *S. maltophilia* in *P. aeruginosa* co-cultures treated with imipenem highlights the potential indirect effects of antibiotic resistance on microbial ecology in polymicrobial lung infections. Our findings demonstrate that antibiotic treatments can indirectly harm resistant community members by altering the strength of interference competition between interacting species. Crucially, change in this competitive interaction was driven by the evolution of imipenem resistance by *P. aeruginosa*, which allowed its survival in the presence of imipenem, leading to the increased production of cytotoxic pyocyanin. Somewhat paradoxically, evolution of *P. aeruginosa* resistance led to the disruption of ecological antibiotic exposure protection provided by *S. maltophilia* through its detoxification of the environment. Antibiotic exposure protection could thus be a transient, evolutionary unstable strategy, similar to facilitative biofilm formation mediated by division of labour [102]. In the context of CF, our findings might help to explain the rapid species turnover typically observed during antibiotic therapy [35], where antibiotic exposure could promote interference competition between resistant community members. This may help explain changes in the relative abundance of resistant species in vivo and why we commonly observe ecological succession and the dominance of individual species within CF microbial communities despite intrinsic resistance to anti-pseudomonal drugs. While such eco-evolutionary dynamics and feedbacks have been observed across multiple systems [52], they warrant further study in the context of CF communities. Furthermore, the two-species in vitro model used here likely represents only a fraction of the genetic and phenotypic diversity present in chronic CF lung populations at both the species and within-species level [103–105]. Expanding clonal and species diversity is hence required to fully understand the role inter-species interactions have in the evolution of antibiotic resistance.

In conclusion, here we demonstrate that while *S. maltophilia* can reduce the efficacy of imipenem therapy at the ecological timescale, this facilitation collapses at the evolutionary timescale. Crucially, *P. aeruginosa* resistance evolution triggered the competitive exclusion of *S. maltophilia* via imipenem-induced production of cytotoxic pyocyanin. Up to a quarter of CF patients can have simultaneous *P. aeruginosa*-*S. maltophilia* co-infections [106, 107], which are often associated with increased mortality [108, 109]. Our findings should hence be set in the context of clinical infection in the future, to better understand the significance of bacterial eco-evolutionary dynamics during antibiotic treatments within patients.

## Materials and methods

### Bacterial strains

We used the *Pseudomonas aeruginosa* PA01:rfp strain as our target pathogen, which was previously constructed by inserting the red fluorescent dTomato gene into the attTn7 site of wild type PA01 by electroporation using the *E. coli* str-DH5α pUC18T-mini-Tn7T-dsRedExpress plasmid [110]. Non-transformed *Pseudomonas aeruginosa* PA01 was also used as an analogous non-fluorescing variant for competition assays. *Stenotrophomonas maltophilia*−518951 strain (SM-518951) was isolated from a sputum sample taken from the respiratory tract of a Danish adult with CF suffering from chronic infection. The sputum sample was provided by Sören Molin and Helle Krogh Johansen at the Rigshospitalet Copenhagen [111]. The strain identity was confirmed by 16s rRNA and whole genome sequencing. Optical density at 595 nm (OD595) and red fluorescence intensity (RFI) at excitation:emission 532:588 nm were used as an estimate of PA01:rfp bacterial growth using the Tecan Infinite 200 plate reader.

### Growth media

We used synthetic cystic fibrosis medium (SCFM) as the culture media because it replicates the average chemical composition of CF lung sputum and can be supplemented with porcine mucin to include the key structural mucous component. SCFM was prepared fresh on the day of use from filter sterilised stock solutions of its individual components following concentrations established previously [53]. The pH was adjusted to between 6.95 and 7.05 using NaOH before vacuum filter sterilisation through 0.22 µm filters. Five g/L porcine mucin was added to simulate mucous concentrations of the CF lung, based on previous studies conducted using SCFM [112] and artificial sputum media (ASM) [113], alongside medical reports [114]. Mucin was pre-sterilised in a 70% ethanol wash and left for 24 h in a 70 °C water bath for the ethanol to evaporate. Mucin was then combined with 100 ml sterile water and left at 4 °C for 24 h with rolling-shaking to dissolve before being aseptically combined with filtered SCFM. *Pseudomonas* isolation agar was made using C-N *Pseudomonas* supplement (100.0 mg cetrimide, 7.5 mg nalidixic acid) according to the manufacturer’s instructions (Oxoid). Inoculation cultures were prepared by streaking frozen bacterial glycerol stocks onto SCFM-agar plates before single colonies were picked and grown overnight (16 h) in 200 μl SCFM media at 37 °C (220 rpm, 12.5 ø). Liquid and agar lysogeny broth (LB) was used when determining *P. aeruginosa* and *S. maltophilia* susceptibility to pyocyanin. Assays involving SCFM used glycerol stocks and inoculation cultures grown and diluted in SCFM and SCFM-agar.

### Determining imipenem degradation by *S. maltophilia* in SCFM

Degradation of imipenem by *S. maltophilia* was quantified by growing bacteria in imipenem treated SCFM for different lengths of time and measuring the growth of inoculated *P. aeruginosa* in the sterilised filtrates. To this end, 20 ml of SCFM containing either 0, 1, 4 and 8 μg/ml imipenem was inoculated with either PA01:rfp, SM-518951, or no bacteria, resulting in 12 treatments in total. The “no imipenem” treatments controlled for any potential effects caused by the consumption of nutrients or the production of secondary metabolites within the media. This experimental design allowed us to determine how *P. aeruginosa* growth was constrained at different imipenem concentrations, and to test if the antimicrobial activity of imipenem was affected by natural degradation (no bacteria inoculated), prior killing of *P. aeruginosa* (PA01:rfp inoculated) or detoxification by *S. maltophilia* (SM-518951 inoculated). *S. maltophilia*’s capacity to degrade imipenem was previously confirmed using liquid chromatography-mass spectrometry [10]. Tubes were incubated at 37 °C with constant shaking for 48 h (220 rpm, 12.5 ø). Every 8 h (8, 16, 24, 32, 40 and 48 h), two ml of media was sampled aseptically, syringe filtered through 0.22 μm filters and divided into six wells on 96-well microplates (185 μl per well). Each well was inoculated with 15 μl PA01:rfp grown in SCFM, creating ~3.75 × 10^5^ cells/ml, and incubated for 24 h. *P. aeruginosa* growth was determined as optical density (OD595 nm).

### Experimental evolution of *P. aeruginosa* in the absence and presence of S. maltophilia in different imipenem antibiotic concentrations

PA01:rfp and SM-518951 were evolved as mono- and co-cultures in the absence (0 μg/ml) and presence of three imipenem concentrations (1, 4 and 8 μg/ml) for a total of 24 days using experimental evolution (8 treatments). These imipenem concentrations were chosen as they reflect the typical clinical concentrations observed in lung sputum during conventional IV [115] and nebulised treatments [116, 117]. Each treatment was replicated ten times, resulting in 80 independent populations. Starting populations contained equal volumes of *P. aeruginosa* and *S. maltophilia* in both monocultures and co-cultures (~3.75 × 10^5^ cells/ml per species) inoculated in 200 μl of SCFM. Microplates were incubated throughout the experiment at 37 °C with constant shaking (220 rpm, 12.5 ø). Every two days, 15 μl of each evolved population was serially transferred into 185 μl of fresh SCFM within corresponding treatments. Imipenem stock solution was prepared on the morning of the transfer by dissolving in SCFM, before being filter sterilised through 0.22 nm filters. To minimise the loss of mucin by filtration only 12.5% of the final SCFM media consisted of the imipenem-containing stock SCFM. Moreover, to control for this effect 0 μg/ml treatments were prepared using 12.5% filter sterilised SCFM. Plate positions and plate number for monoculture and co-culture replicates were randomised at each serial transfer. All evolved bacterial populations were cryopreserved at −80 °C in 25% glycerol at days 4, 12, 18 and 24. *P. aeruginosa* population densities were quantified as red fluorescence intensity (RFI) every 2 days (excitation:emission 532:588 nm).

### Detecting the presence of *S. maltophilia* in co-cultures

The presence of *S. maltophilia* in co-culture populations was evaluated by plating undiluted glycerol stocks directly onto 64 μg/ml tobramycin containing SCFM-agar plates and grown for 24 h at 37 °C. *S. maltophilia* was considered alive and present if there was any observable growth of the white dot-like colonies characteristic of *S. maltophilia*, which was intrinsically resistant to high concentrations of tobramycin, while evolved *P. aeruginosa* isolates were susceptible.

### Isolation of *P. aeruginosa* colonies from evolved populations

Evolved *P. aeruginosa* colonies were isolated by serially diluting and spreading frozen glycerol stocks of replicate populations onto *Pseudomonas*-selective SCFM-agar plates. Bacteria were grown for 16 h at 37 °C before a single colony from each individual replicate population was randomly selected and grown for 16 h in 200 μl SCFM at 37 °C with shaking (220 rpm, 12.5 ø), before cryopreserving at −80 °C in 25% glycerol. Evolved bacteria were isolated from each replicate population at days 4, 12, 18 and 24 of the selection experiment, resulting in a total of 79 isolates per time point as PA01 went extinct by day 4 in one of the 8 μg/ml *P. aeruginosa* monoculture populations (this replicate was hence excluded from the analyses). Moreover, only 63 of 80 populations were used for analysing the day 4 time point due to accidental loss of replicates 9 and 10 in all treatments.

### Quantifying imipenem resistance of evolved *P. aeruginosa* isolates

The imipenem resistance of all evolved *P. aeruginosa* isolates from all time points (*n* = 300) was determined using microdilution assays in SCFM. Briefly, twelve concentrations (0–128 μg/ml), with the middle concentration determined by the clinical breakpoint for *P. aeruginosa* [118], were inoculated to ~3.75 × 10^5^ cells/ml. Cell densities were standardised by diluting overnight growth cultures in SCFM and imipenem solutions were prepared as previously described. Plates were grown for 24 h at 37 °C with constant shaking (220 rpm, 12.5 ø) and bacterial growth was determined as optical density (OD595 nm). The assay row for each clone was randomised within replicates alongside plate positions. Antibiotic resistance was determined as bacterial growth in concentrations above the clinical breakpoint of 2 μg/ml [118] and the minimum inhibitory concentration (MIC) was determined as the lowest imipenem concentration with no observable growth relative to uninoculated SCFM. MIC assays were repeated twice for each clone.

### Evaluating changes in competitive fitness of evolved *P. aeruginosa* isolates

Changes in the competitive fitness of evolved isolates from day 24 was quantified by performing direct competition assays against the non-fluorescently tagged, otherwise isogenic PA01 ancestor in liquid SCFM. In these assays, SCFM was filtered after the addition of 5.0% mucin to enable more precise growth measurements. Overnight cultures of all evolved isolates were prepared as described earlier; 15 μl of evolved isolates and 15 μl of PA01 ancestor (~5.35 × 10^5^ cells ml^−1^ for each) were inoculated in 170 μl SCFM on 96-well microplates and incubated at 37 °C for 22 h with constant shaking (220 rpm, 12.5 ø). The OD595 and RFI readings were taken after 24 h and the background OD595 and RFI values of uninoculated SCFM were subtracted. Since the intensity of fluorescence depends on the total abundance of cells, RFI scores were normalised against total culture growth (RFI/OD595) to give the relative competitive success of evolved isolates in each individual culture. At least ten technical replicates were performed for each evolved clone and averaged to give a single measurement of fitness. Well positions for each clone were randomised and two replicate groups per evolved clone were measured on independent microwell plates. Ancestral PA01 and PA01:rfp strains were included on all measurement plates to control for between-plate variation. The coefficient of variation between ancestral strain replicates across plates was 0.0715, demonstrating minimal growth variation among plates. Competitive index scores of evolved isolates were standardised relative to the average competitive score of the ancestral PA01:rfp strain.

### Measuring the production of pyoverdine in *P. aeruginosa*

The production of pyoverdine in evolved PA01:rfp isolates (day 24) and the ancestral PA01:rfp was measured by incubating cultures for 48 h at 37 °C with constant shaking (220 rpm, 12.5 ø). Pyoverdine is a virulence factor commonly produced by *P. aeruginosa* during CF lung infection [119, 120] that is involved in iron sequestration and indirect competitor inhibition [57, 58]. OD595 (nm) readings were then taken before cultures were centrifuged at 725 × *g* at room temperature for 15 min. Supernatants were filtered and centrifuged again for a further 15 min at 200 × *g* before OD 405 and 595 nm measurements taken. Supernatants exceeding an OD595 reading of 0.15 indicated pellet dispersal and were excluded from the analysis and repeated.

### Measuring direct inhibition of *S. maltophilia* by *P. aeruginosa* when pre-grown in the absence or presence of imipenem

To test if imipenem exposure induced *S. maltophilia* inhibition by *P. aeruginosa*, eight evolved PA01:rfp isolates from 4 µg/ml and 8 µg/ml imipenem treatment populations evolved both in the absence or presence of *S. maltophilia* (day 24) were assayed alongside the ancestral PA01:rfp strain. All isolates were grown in the absence and presence of imipenem (4 and 8 µg/ml) in SCFM media at 37 °C for 48 h. After which, 2 µl of bacterial cultures were spotted onto soft SCFM-agar lawns containing ancestral *S. maltophilia* overnight cultures. Plates were then incubated at 37 °C for 48 h and pictures taken every 24 h at a standardised distance and inhibition zones determined as the inhibition halo surrounding *P. aeruginosa* colonies. The zone of *S. maltophilia* inhibition was measured using imageJ (version: 1.53) after converting images to 8-bit (grey-scale) and enhancing light–dark contrast. An image threshold value of 12% was used to distinguish the inhibition zone, which was measured based on Feret diameter.

### Measuring the effect of imipenem exposure on pyocyanin production in *P. aeruginosa*

The production of pyocyanin, a cytotoxic virulence factor involved in direct competitor killing [61, 62] that is associated with worsened pulmonary pathophysiology [121], was quantified as previously described [122, 123]. Briefly, SCFM cultures with 0, 4 and 8 µg/ml imipenem were inoculated with eight evolved imipenem-resistant *P. aeruginosa* isolates from 4 and 8 µg/ml treated imipenem populations (day 24; with and without *S. maltophilia*), alongside the ancestral PA01:rfp strain on 96-well microplates. Cultures were incubated for 48 h at 37 °C with constant shaking (220 rpm, 12.5 ø) before measuring cell density (OD595 nm). Plates were then centrifuged at 725 × *g* at room temperature for 15 min before 150 µl of supernatant from all wells were transferred to 96-well filter microplates (Agilent). Supernatants were filtered using centrifugation for a further 15 min at 200 × *g* and OD measured at wavelength 595 and 695 nm. Supernatants exceeding an OD595 reading of 0.15 indicated pellet dispersal and were repeated. Relative pyocyanin production was standardised with cell densities by dividing the OD695 values of the filtered supernatant with the OD595 of the non-filtered cultures. Estimates of the average concentration of pyocyanin produced by imipenem-resistant *P. aeruginosa* isolates in the presence and absence of imipenem was extrapolated from pyocyanin calibration curves (*n* = 8) between concentrations of 0 to 32 µg/ml. The background OD695 of the 0 µg/ml pyocyanin concentration was subtracted from the calibration curve before comparison, while the average background OD695 of uninoculated blank supernatants (*n* = 28) was subtracted from the *P. aeruginosa* supernatant pyocyanin measurements.

### Quantifying the cytotoxicity of pyocyanin for the growth of *S. maltophilia* and *P. aeruginosa*

The cytotoxicity of pyocyanin was evaluated using microdilution assays in liquid LB media. *P. aeruginosa*-derived pyocyanin crystals (Sigma-Aldrich) were dissolved in Dimethyl sulfoxide (DMSO) and diluted to a starting concentration of 50 µg/ml in LB media (5% DMSO). A further nine 50% dilution steps were prepared on microplate wells using serial dilution alongside non-pyocyanin LB and DMSO-LB (5%) control treatments. Four replicate wells of each treatment concentration were then inoculated with 50 µl overnight culture of *S. maltophilia* or PA01:rfp diluted by 1:10 in LB media, creating final pyocyanin concentrations between 33.3 and 0.065 µg/ml (Fig. 5C). Microplates were incubated for 48 h without shaking and bacterial densities quantified as optical density (OD595 nm) at 24 and 48 h time points. After 48 h, 0.2 µl of all cultures were spotted onto LB agar plates and bacterial survival determined as the detectable growth of bacterial colonies after 48 h of incubation at 37 °C. Four replicates were performed for each bacterial strain.

### Sequencing and genomic analysis of evolved *P. aeruginosa* isolates

In total, 48 evolved *P. aeruginosa* colonies were isolated and sequenced from the final time point of the selection experiment (24 days). Six independent replicate populations per treatment were randomly selected, and one clone per replicate was sequenced alongside the ancestral PA01:rfp strain. Genome sequencing was provided by MicrobesNG using the following protocol: Three beads were washed with extraction buffer containing lysozyme and RNase A, incubated for 25 min at 37 °C. Proteinase K and RNaseA were added and incubated for 5 min at 65 °C. Genomic DNA was purified using an equal volume of SPRI beads and resuspended in EB buffer. DNA was quantified in triplicates with the Quantit dsDNA HS assay in an Ependorff AF2200 plate reader. Genomic DNA libraries were prepared using Nextera XT Library Prep Kit (Illumina, San Diego, USA) following the manufacturer’s protocol with the following modifications: two nanograms of DNA instead of one were used as input, and PCR elongation time was increased to 1 min from 30 s. DNA quantification and library preparation were carried out on a Hamilton Microlab STAR automated liquid handling system. Pooled libraries were quantified using the Kapa Biosystems Library Quantification Kit for Illumina on a Roche light cycler 96 qPCR machine. Libraries were sequenced on an HiSeq/NovaSeq (Illumina) instrument using a 250 bp paired end protocol. Reads were adapter trimmed using Trimmomatic (version: 0.30) with a sliding window quality cut-off of Q15 [124]. De novo assembly was performed on samples using SPAdes (version: 3.7) [125], and contigs were annotated using Prokka (version: 1.11) [126].

Genome data was analysed as follows: SNP’s, indels and chromosomal rearrangements were identified by assembling the paired-fastq reads of sequenced isolates against the published PA01 reference genome “AE004091.2”. Chromosomal mutations and their predicted effects were identified using Breseq (version: 0.35.4) [127]. In total, 28 variants were identified out of 48 sequenced isolates, containing 41 mutations (average 0.85 per clone), 36 of which were non-synonymous leaving five synonymous mutations including those in intergenic regions. Mutations identified in the ancestral PA01:rfp were filtered out and excluded from further analyses. The de novo mutations that emerged in the selection experiment were verified visually using the integrative genomic viewer (version: 2.8.10) [128, 129].

### Statistical analysis

To compare how the presence of *P. aeruginosa* and *S. maltophilia* impacted the rate of imipenem degradation, area under the curve (AUC) measurements were calculated from PA01:rfp’s growth in sterile filtrate plotted against sampling time point from the original culture (Fig. 1A–D). These AUC scores provided a proxy for *P. aeruginosa*’s overall growth capacity in imipenem treated media over the 48 h period. The growth AUC variable was analysed using Analysis of Variance (ANOVA), comparing the effect of *S. maltophilia* and no-bacteria treatments on change in media habitability, excluding the *P. aeruginosa* treatment and including media imipenem treatment concentration as a model effect. All ANOVA models used throughout this study utilised standard least squares with an emphasis on effect leverage. AUC measurements were calculated using the trapezium rule, taking the integral of the combined areas between each successive time point. Welch’s t-tests, assuming unequal variance, were used to compare 48 h AUC scores from *S. maltophilia* and *P. aeruginosa* treatments against the no-bacteria control within individual imipenem concentrations. The impact of *S. maltophilia* presence and well imipenem concentration on the growth of PA01:rfp during MIC assays, measured in RFI, was analysed using a full-factorial ANOVA, with imipenem concentration transformed on a log2 scale. The interaction term between *S. maltophilia* presence and imipenem well concentration was used to evaluate if *S. maltophilia* protection was more pronounced at higher imipenem concentrations.

To evaluate the statistical significance of evolved imipenem resistance in *P. aeruginosa*, the average MIC and area under the MIC curve (MIC AUC) scores of isolates within each imipenem selection concentration (0, 1, 4 and 8 μg/ml) was analysed with full-factorial ANOVA, including sampling time point and *S. maltophilia* presence as factors. A significant effect of sampling time point indicated a significant change in imipenem resistance over the course of the selection experiment while the interaction term evaluated if *S. maltophilia* had any significant effect on the rate of imipenem resistance evolution. ANOVA was also used to analyse the correlation between the emergence of imipenem resistance and the detectable presence of *S. maltophilia* in co-cultures treated with 4 and 8 μg/ml imipenem. Once *S. maltophilia* went extinct from a population, isolates from subsequent sampling time points were excluded to avoid biasing the analysis against the null hypothesis. Changes in competitive fitness were analysed with Dunnett’s tests for each imipenem selection concentration, using ancestral replicates as the control group (*n* = 12). The relationship between imipenem resistance and competitive fitness was determined using multiple linear regression, including evolved presence or absence of *S. maltophilia* as both a factor and as an interaction effect with competitive fitness. Evolved imipenem culture concentration was excluded due to high correlation with level of evolved imipenem resistance. Homoscedasticity of models was evaluated using Levene’s test. For the competitive fitness data this assumption was invalidated (*p* = 0.015) and a log10 transformation was performed (*p* = 0.083) before ANOVA and Dunnett’s test analysis. Mean difference (MD) scores between treatment groups and related graphs used untransformed data for ease of interpretation.

Kaplan–Meier survival curves were used to evaluate the rate of *S. maltophilia* extinctions in co-culture populations during the selection experiment with significance determined using the log-rank test. Inhibition zone and pyocyanin data were separately analysed using restricted maximum likelihood (REML) based mixed model analysis, using isolate number as a random effect and evolved imipenem treatment, evolved presence or absence of *S. maltophilia* and the experimental imipenem treatment concentration, as fixed effects. The degree to which covariance across isolates explained variation within treatment fixed effects was evaluated using Wald’s test. Dunnett’s tests were used to compare evolved versus ancestral phenotypes in the absence of imipenem. Throughout this study, data handling was done in excel (version: 2009). Statistical analyses and initial graph drawing were performed in R (version: 4.0.1). Graphics were visually enhanced in Inkscape (version: 1.0.1).

## Supplementary information

Supplementary Information

## Data Availability

Raw data is available on FigShare (10.6084/m9.figshare.19175330.v1) and sequence data is uploaded to NCBI (PRJNA788460).
