# Supplementary material for: Resistance evolution can disrupt antibiotic exposure protection through competitive exclusion of the protective species
Source: ISME J. 2022 Jul 20;16(10):2433–47. doi: 10.1038/s41396-022-01285-w (PMC9477885; doi:10.1038/s41396-022-01285-w)
Supplement: Supplementary file 1 — Supplementary Information [file 41396_2022_1285_MOESM1_ESM.pdf]

Resistance evolution can disrupt antibiotic exposure protection through competitive exclusion of the protective species

Angus M. Quinn[1,2], Michael J. Bottery[2,3], Harry Thompson[2] & Ville-Petri Friman[2]

[1]School of Life Sciences, University of Warwick, Coventry, CV4 7AL, UK.

[2]Department of Biology, University of York, Wentworth Way, York, YO10 5DD, UK.

[3]Division of Evolution, Infection and Genomics, School of Biological Sciences, University of Manchester, Manchester, M13 9PL, UK.

Supplementary Information

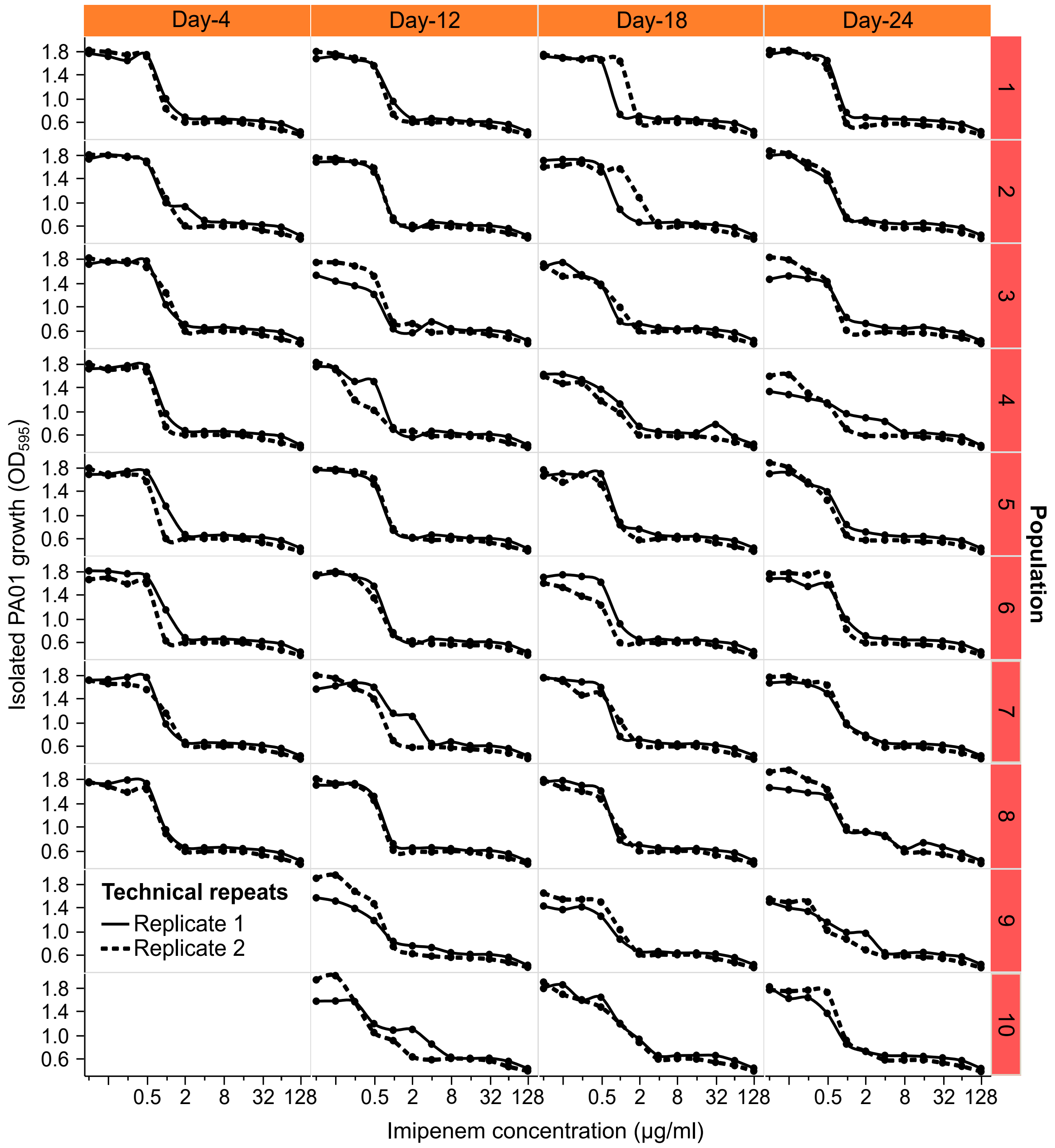

SI figure 1. **MIC curves of monocultured *P. aeruginosa* evolved in 0 µg/ml imipenem.** The imipenem resistance of PA01:rfp isolates evolved in independent monoculture populations in the absence of imipenem was measured using minimum inhibitory concentration (MIC) assays across two technical replicates (solid & dotted; N=1).

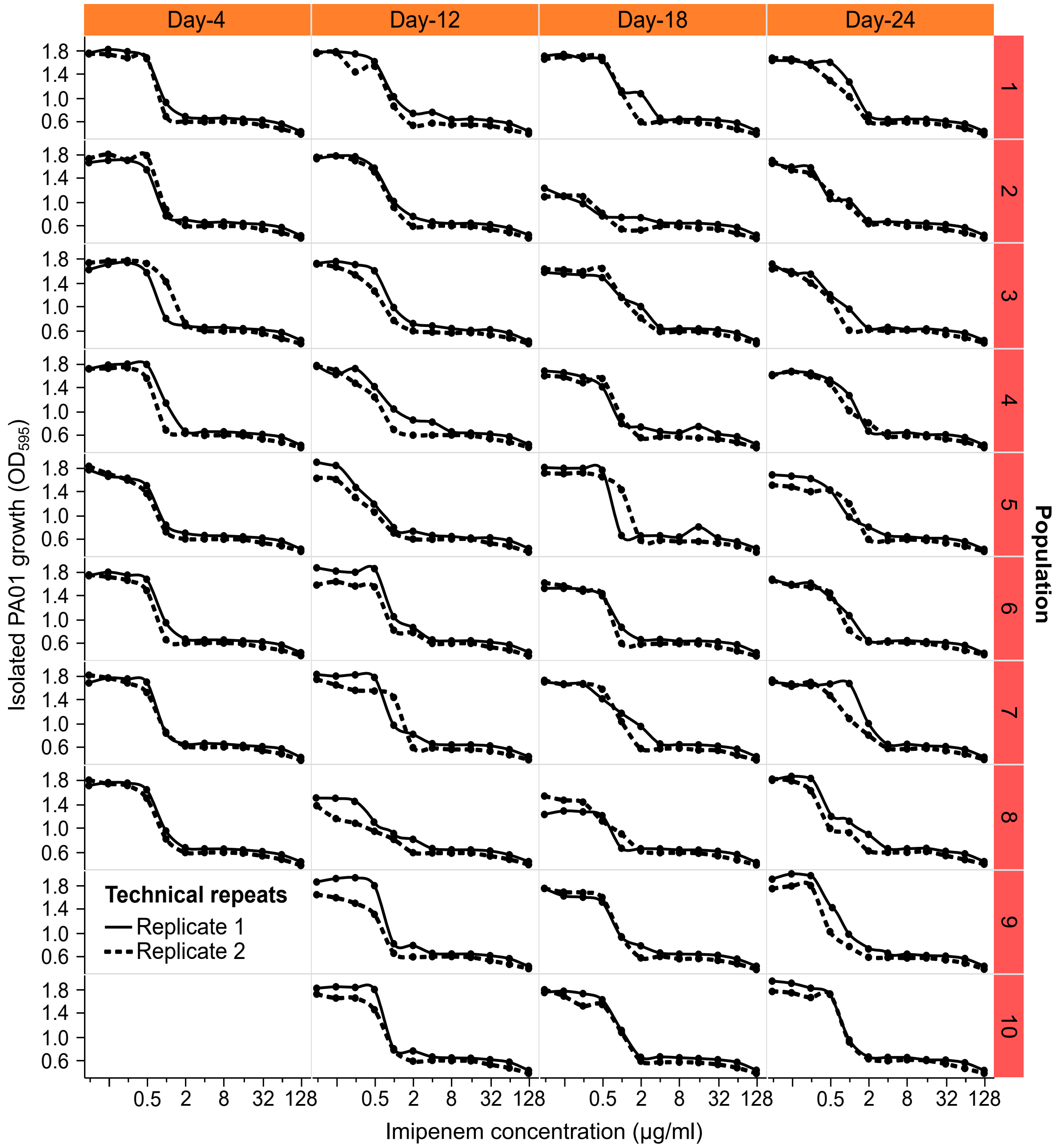

SI figure 2. **MIC curves of *S. maltophilia* co-cultured *P. aeruginosa* evolved in 0 μg/ml imipenem.** The imipenem resistance of PA01:rfp isolates evolved in independent *S. maltophilia* co-culture populations in the absence of imipenem was measured using minimum inhibitory concentration (MIC) assays across two technical replicates (solid & dotted; N=1).

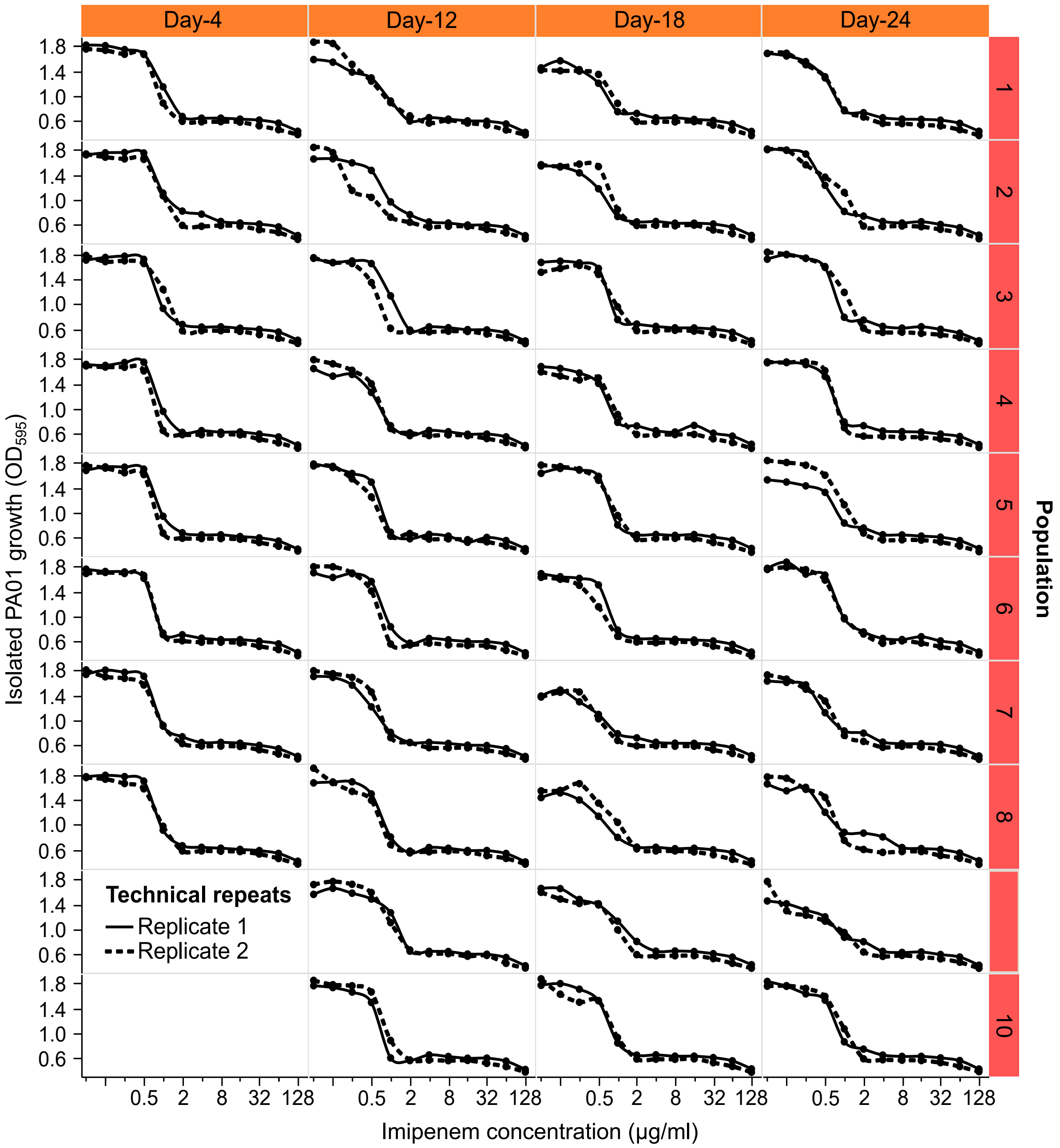

SI figure 3. **MIC curves of monocultured *P. aeruginosa* evolved in 1 µg/ml imipenem.** The imipenem resistance of PA01:rfp isolates evolved in independent monoculture populations in the presence of 1 µg/ml imipenem was measured using minimum inhibitory concentration (MIC) assays across two technical replicates (solid & dotted; N=1).

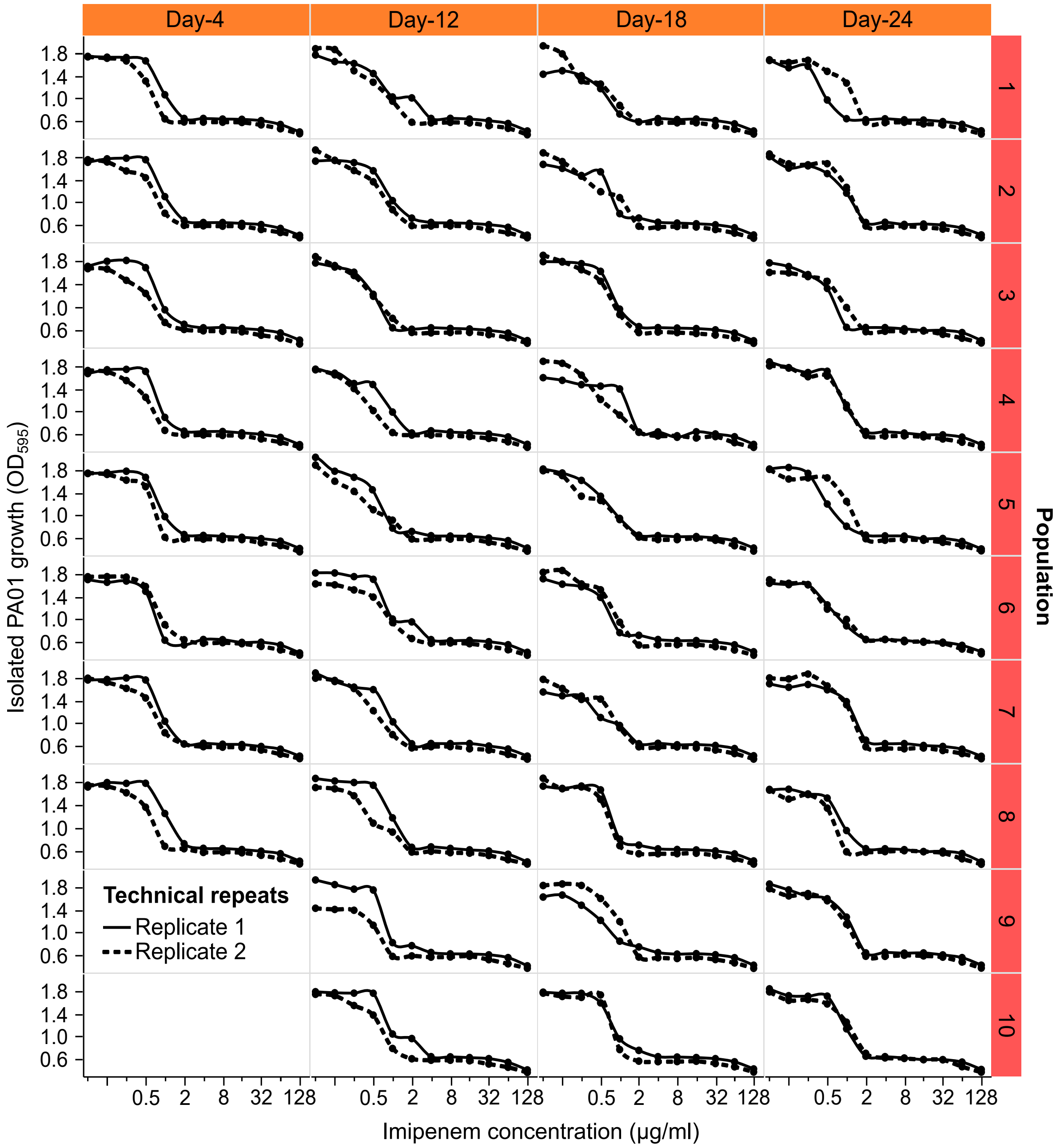

SI figure 4. **MIC curves of *S. maltophilia* co-cultured *P. aeruginosa* evolved in 1 µg/ml imipenem.** The imipenem resistance of PA01:rfp isolates evolved in independent *S. maltophilia* co-culture populations in the presence of 1 µg/ml imipenem was measured using minimum inhibitory concentration (MIC) assays across two technical replicates (solid & dotted; N=1).

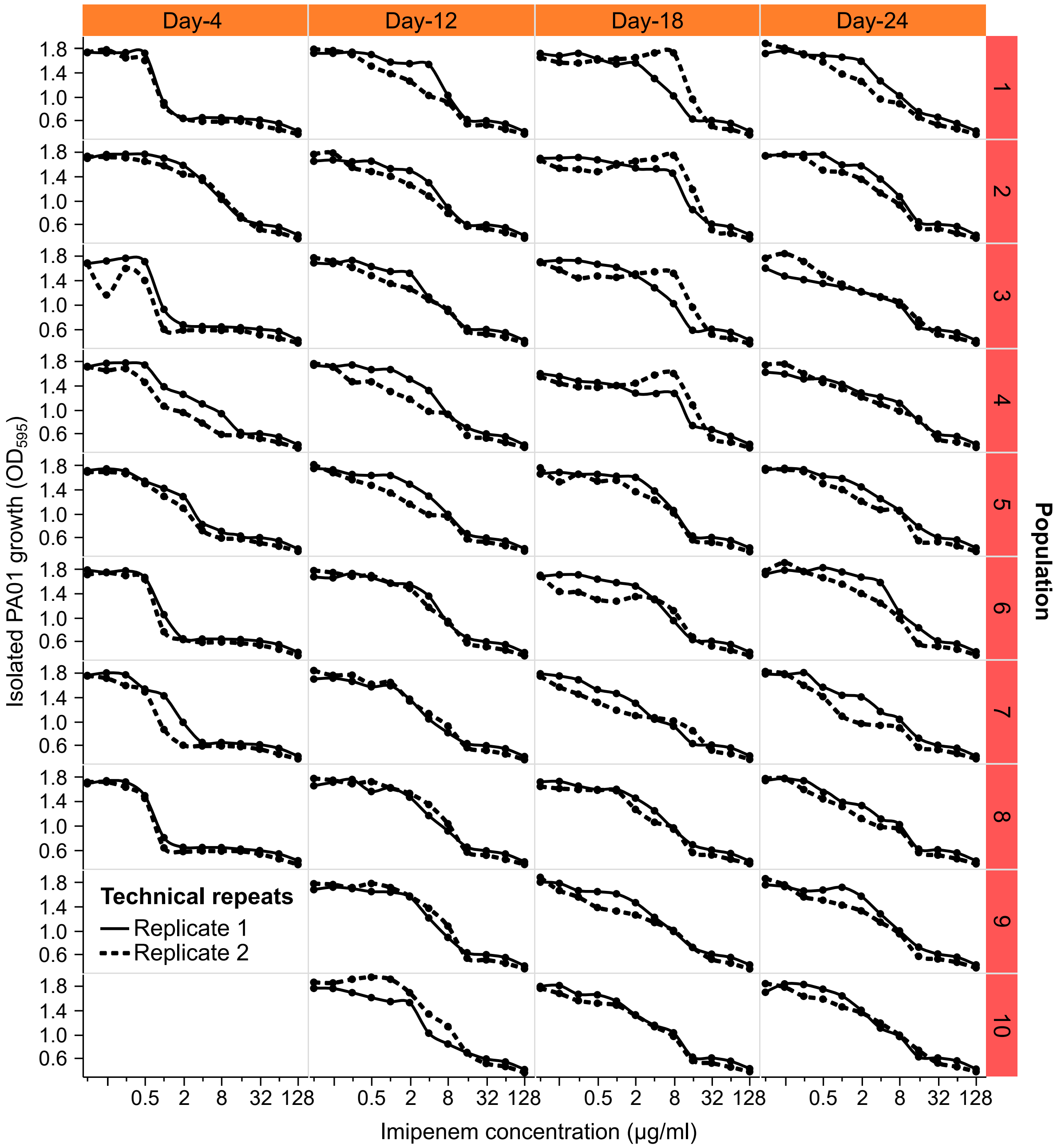

SI figure 5. **MIC curves of monocultured *P. aeruginosa* evolved in 4 µg/ml imipenem.** The imipenem resistance of PA01:rfp isolates evolved in independent monoculture populations in the presence of 4 µg/ml imipenem was measured using minimum inhibitory concentration (MIC) assays across two technical replicates (solid & dotted; N=1).

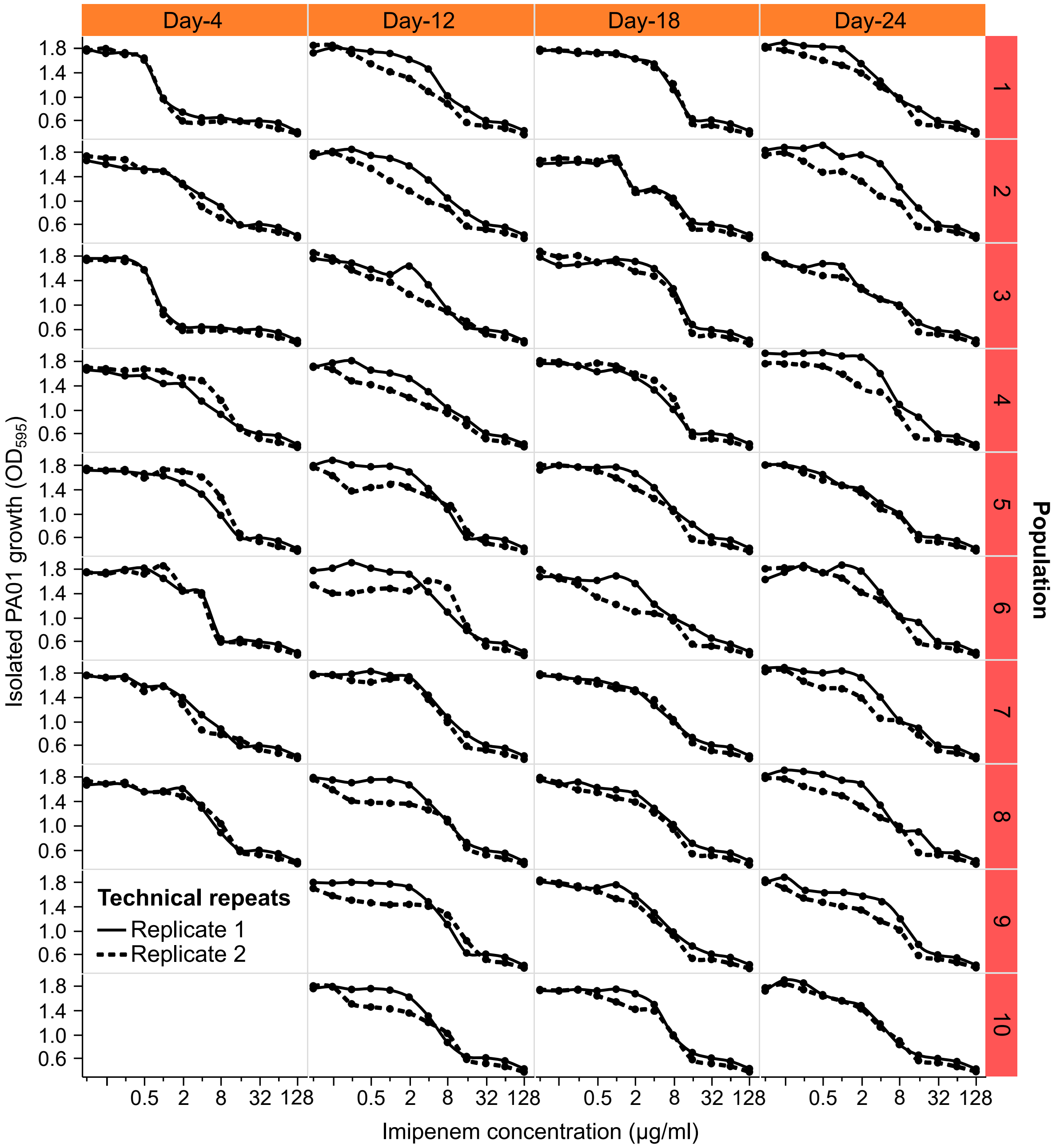

SI figure 6. **MIC curves of *S. maltophilia* co-cultured *P. aeruginosa* evolved in 4 µg/ml imipenem.** The imipenem resistance of PA01:rfp isolates evolved in independent *S. maltophilia* co-culture populations in the presence of 4 µg/ml imipenem was measured using minimum inhibitory concentration (MIC) assays across two technical replicates (solid & dotted; N=1).

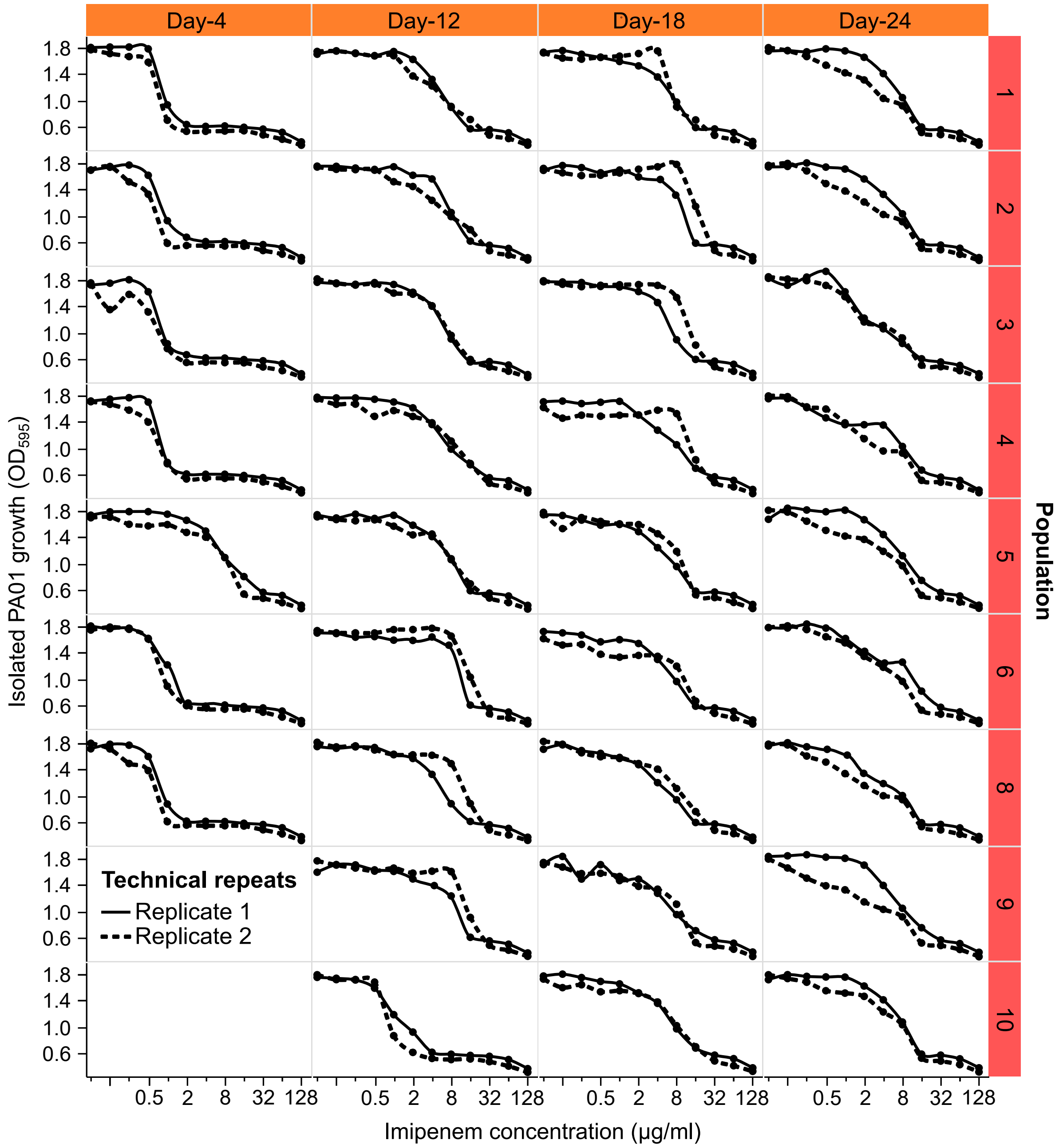

***SI figure 7* – MIC curves of monocultured *P. aeruginosa* evolved in 8 µg/ml imipenem.** The imipenem resistance of PA01:rfp isolates evolved in independent monoculture populations in the presence of 8 µg/ml imipenem was measured using minimum inhibitory concentration (MIC) assays across two technical replicates (solid & dotted; N=1).

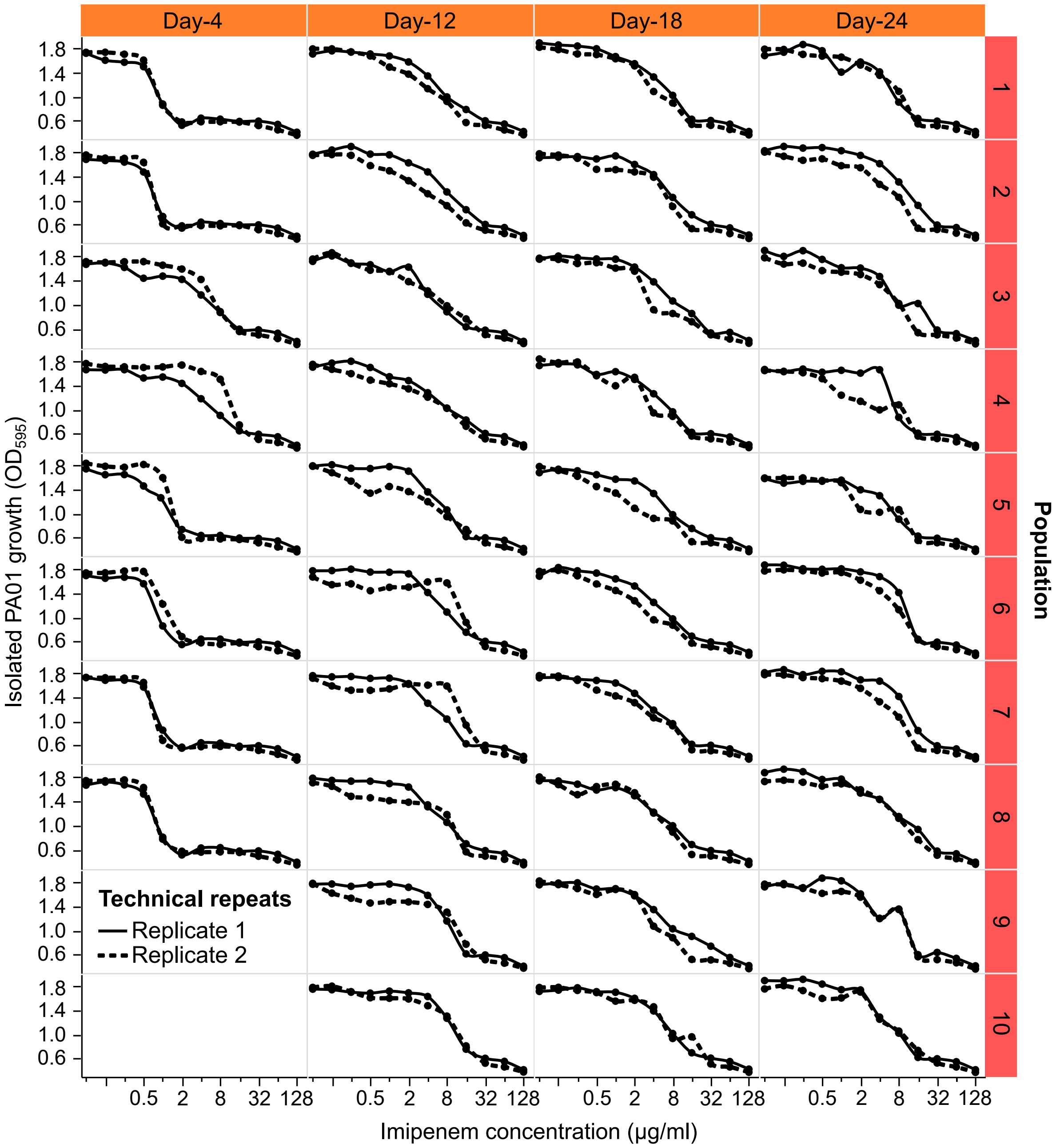

SI figure 8. **MIC curves of *S. maltophilia* co-cultured *P. aeruginosa* evolved in 8 μg/ml imipenem.** The imipenem resistance of PA01:rfp isolates evolved in independent *S. maltophilia* co-culture populations in the presence of 8 μg/ml imipenem was measured using minimum inhibitory concentration (MIC) assays across two technical replicates (solid & dotted; N=1).

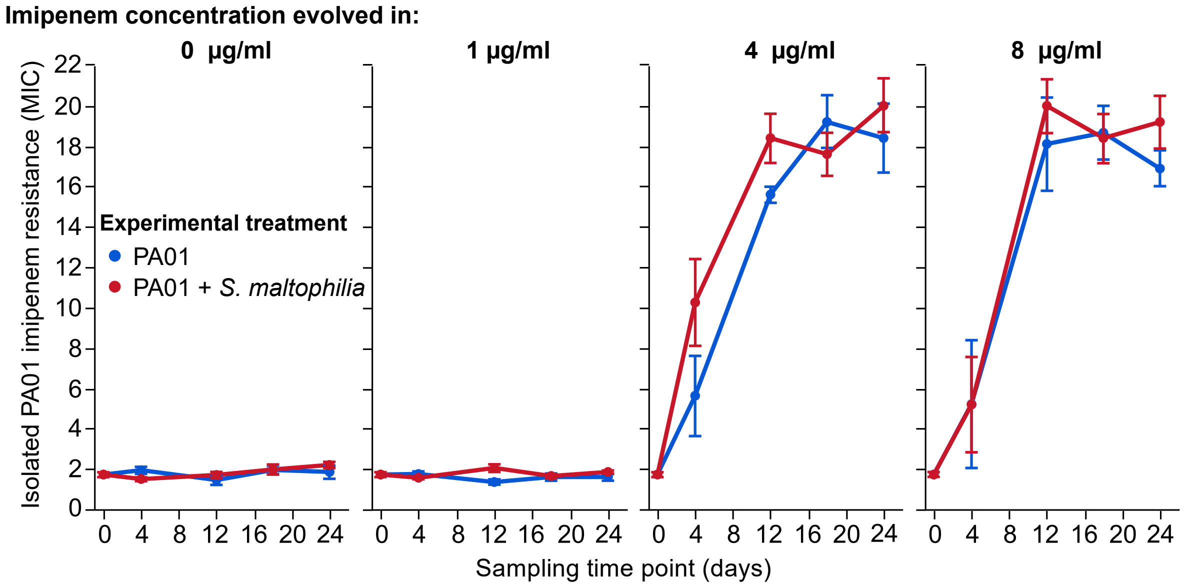

SI figure 9. Minimum inhibitory concentration of evolved *P. aeruginosa* clones evolved in the presence and absence of *S. maltophilia* and imipenem. The imipenem resistance of PA01:rfp isolated from independent monoculture (blue) and *S. maltophilia* co-culture (red) populations evolved in either 0, 1, 4 or 8 µg/ml imipenem was measured using minimum inhibitory concentration (MIC) assays. Two technical replicates were performed per isolate and error bars correspond to ± one standard error of the mean. (N=10).

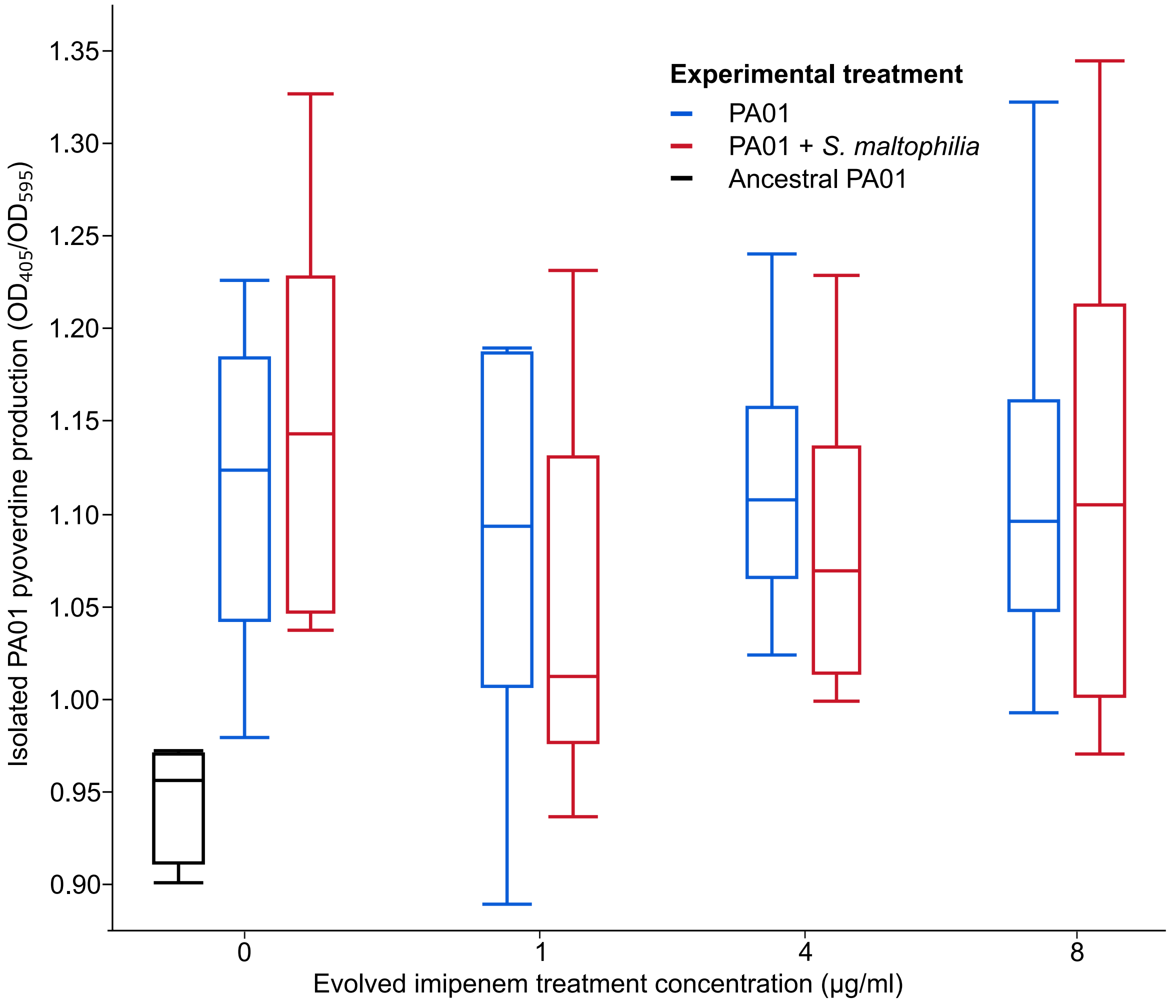

SI figure 10. Pyoverdine production is upregulated in SCFM-evolved *P. aeruginosa*. The production of pyoverdine in PA01:rfp isolated from independent monoculture (blue) and *S. maltophilia* co-culture (red) populations evolved in either 0, 1, 4 or 8 µg/ml imipenem was measured as optical density (405 nm) and normalised against the bacterial growth (595 nm) in the pre-centrifuged cultures (OD405/OD595). Tukey boxplots represent the interquartile range (25th-75th percentile), whiskers show the minimum and maximum values, and the midline shows the median value (N=10; Ancestral: N=12).

SI table 1. Mutations of *P. aeruginosa* evolved in SCFM in the presence and absence of *S. maltophilia* and imipenem (page 1)

Organism	Accession #	Seq ID	Evolved Imipenem (μg/ml)	Evolved with *S. maltophilia*	Replicate Population	Imipenem Resistant	Mutation Position (nt)	Mutation Outcome	Mutation Effect	Annotation	Gene
PA01:dtomato	SAMN24000132	31881	0	No	9	No	2,449,917	Δ3,332 bp	Deletion	na	*PA2228–30*
PA01:dtomato	SAMN24000133	31882	0	No	2	No	4,300,010	C→G	Intergenic	(+12/-107)	*PA3838/9*
PA01:dtomato	SAMN24000134	31883	0	No	8	No	No mutations				
PA01:dtomato	SAMN24000135	31884	0	No	1	No	No mutations				
PA01:dtomato	SAMN24000136	31885	0	No	4	No	No mutations				
PA01:dtomato	SAMN24000137	31886	0	No	6	No	No mutations				
PA01:dtomato	SAMN24000139	31887	0	Yes	5	No	No mutations				
PA01:dtomato	SAMN24000139	31888	0	Yes	2	No	786,570	G→A	AA Substitution	C201Y (TGT→TAT)	*PA0715*
PA01:dtomato	SAMN24000139	31888	0	Yes	2	No	786,647	A→G	AA Substitution	K227E (AAA→GAA)	*PA0715*
PA01:dtomato	SAMN24000139	31888	0	Yes	2	No	3,605,587	C	Intergenic	(+120/-97)	*PA3216/cyaB*
PA01:dtomato	SAMN24000139	31888	0	Yes	2	No	4,843,878	C	AA Substitution	S23P (TCC→CCC)	*mvaT*
PA01:dtomato	SAMN24000139	31888	0	Yes	2	No	5,069,342	C	AA Substitution	S64G (AGC→GGC)	*pilA*
PA01:dtomato	SAMN24000140	31889	0	Yes	9	No	5,070,925	A→C	AA Substitution	D388A (GAC→GCC)	*pilB*
PA01:dtomato	SAMN24000140	31889	0	Yes	9	No	2,039,331	G→C	AA Substitution	G964A (GGC→GCC)	*PA1874*
PA01:dtomato	SAMN24000140	31889	0	Yes	9	No	2,039,326	A→C	Synonymous	T962T (ACA→ACC)	*PA1874*
PA01:dtomato	SAMN24000140	31889	0	Yes	9	No	2,039,330	G→T	AA Substitution	G964C (GGC→TGC)	*PA1874*
PA01:dtomato	SAMN24000140	31889	0	Yes	9	No	2,040,563	T→A	AA Substitution	S1375T (TCG→ACG)	*PA1874*
PA01:dtomato	SAMN24000140	31889	0	Yes	9	No	2,040,532	T→C	Synonymous	D1364D (GAT→GAC)	*PA1874*
PA01:dtomato	SAMN24000140	31889	0	Yes	9	No	2,041,075	C→G	Synonymous	T1545T (ACC→ACG)	*PA1874*
PA01:dtomato	SAMN24000141	31890	0	Yes	10	No	No mutations				
PA01:dtomato	SAMN24000142	31891	0	Yes	8	No	No mutations				
PA01:dtomato	SAMN24000143	31892	0	Yes	3	No	No mutations				
PA01:dtomato	SAMN24000144	31893	1	No	3	No	706,182	T→G	AA Substitution	H164P (CAC→CCC)	*vfr*
PA01:dtomato	SAMN24000145	31894	1	No	8	No	No mutations				
PA01:dtomato	SAMN24000146	31895	1	No	7	No	No mutations				
PA01:dtomato	SAMN24000147	31896	1	No	5	No	No mutations				
PA01:dtomato	SAMN24000148	31897	1	No	10	No	No mutations				
PA01:dtomato	SAMN24000149	31898	1	No	6	No	No mutations				
PA01:dtomato	SAMN24000150	31899	1	Yes	7	No	No mutations				
PA01:dtomato	SAMN24000151	31900	1	Yes	8	No	No mutations				
PA01:dtomato	SAMN24000152	31901	1	Yes	5	No	No mutations				

SI table 1. Mutations of *P. aeruginosa* evolved in SCFM in the presence and absence of *S. maltophilia* and imipenem (page 2)

Organism	Accession #	Seq ID	Evolved Imipenem (µg/ml)	Evolved with *S. maltophilia*	Replicate Population	Imipenem Resistant	Mutation Position (nt)	Mutation Outcome	Mutation Effect	Annotation	Gene
PA01:dtomato	SAMN24000153	31902	1	Yes	4	No	No mutations				
PA01:dtomato	SAMN24000154	31903	1	Yes	9	No	No mutations				
PA01:dtomato	SAMN24000155	31904	1	Yes	10	No	No mutations				
PA01:dtomato	SAMN24000156	31905	4	No	8	Yes	1,044,114	$(TGGAC)_{1\to2}$	Frameshift (5 bp insertion)	(1201/1332 nt)	*oprD*
PA01:dtomato	SAMN24000157	31906	4	No	1	Yes	1044612	G→A	Stop Insertion	Q235* (Cag/Tag)	*oprD*
PA01:dtomato	SAMN24000158	31907	4	No	7	Yes	1045040	-G	Frameshift	(274/1332 nt)	*oprD*
PA01:dtomato	SAMN24000159	31908	4	No	5	Yes	1,044,105	$(CGGAC)_{2\to1}$	Frameshift (5 bp deletion)	(1206-1210/1332 nt)	*oprD*
PA01:dtomato	SAMN24000159	31908	4	No	5	Yes	4,524,185	A→C	AA Substitution	Y324D (TAC→GAC)	*PA4041*
PA01:dtomato	SAMN24000160	31909	4	No	9	Yes	1,044,588	-T	Frameshift	(727/1332 nt)	*oprD*
PA01:dtomato	SAMN24000161	31910	4	No	10	Yes	1,951,335	T→G	AA Substitution	T131P (ACC→CCC)	*parS*
PA01:dtomato	SAMN24000161	31910	4	No	10	Yes	1,044,199	-AC	Frameshift	(1115-1116/1332 nt)	*oprD*
PA01:dtomato	SAMN24000162	31911	4	Yes	4	Yes	1,044,109	+G	Frameshift	(1206/1332 nt)	*oprD*
PA01:dtomato	SAMN24000163	31912	4	Yes	3	Yes	1,044,210	-C	Frameshift	(1105/1332 nt)	*oprD*
PA01:dtomato	SAMN24000164	31913	4	Yes	7	Yes	1,044,881	-G	Frameshift	(434/1332 nt)	*oprD*
PA01:dtomato	SAMN24000165	31914	4	Yes	5	Yes	1,044,298	C→T	Stop Insertion	W339* (TGG→TGA)	*oprD*
PA01:dtomato	SAMN24000166	31915	4	Yes	9	Yes	1,044,030	G→A	Stop Insertion	Q429* (CAG→TAG)	*oprD*
PA01:dtomato	SAMN24000167	31916	4	Yes	1	Yes	1,045,116	G→A	Stop Insertion	Q67* (CAA→TAA)	*oprD*
PA01:dtomato	SAMN24000168	31917	8	No	5	Yes	1,045,042	G→T	Stop Insertion	Y91* (TAC→TAA)	*oprD*
PA01:dtomato	SAMN24000169	31918	8	No	2	Yes	1,045,116	G→A	Stop Insertion	Q67* (CAA→TAA)	*oprD*
PA01:dtomato	SAMN24000170	31919	8	No	8	Yes	1,045,121	C→T	Stop Insertion	W65* (TGG→TAG)	*oprD*
PA01:dtomato	SAMN24000171	31920	8	No	9	Yes	1,044,064	C→T	Stop Insertion	W417* (TGG→TGA)	*oprD*
PA01:dtomato	SAMN24000172	31921	8	No	1	Yes	1,044,408-98	Δ91 bp	New Junction	na	*oprD*
PA01:dtomato	SAMN24000173	31922	8	No	4	Yes	1,044,109	+G	Frameshift	(1206/1332 nt)	*oprD*
PA01:dtomato	SAMN24000174	31923	8	Yes	8	Yes	1,044,001	+A	Frameshift	(1314/1332 nt)	*oprD*
PA01:dtomato	SAMN24000175	31924	8	Yes	1	Yes	1,045,116	G→A	Stop Insertion	Q67* (CAA→TAA)	*oprD*
PA01:dtomato	SAMN24000176	31925	8	Yes	3	Yes	1,044,210	-C	Frameshift	(1105/1332 nt)	*oprD*
PA01:dtomato	SAMN24000177	31926	8	Yes	2	Yes	1,044,679	+C	Frameshift	(636/1332 nt)	*oprD*
PA01:dtomato	SAMN24000178	31927	8	Yes	9	Yes	1,044,030	G→A	Stop Insertion	Q429* (CAG→TAG)	*oprD*
PA01:dtomato	SAMN24000179	31928	8	Yes	10	Yes	1,045,103	-G	Frameshift	(212/1332 nt)	*oprD*

SI table 1. Mutations of *P. aeruginosa* evolved in SCFM in the presence and absence of *S. maltophilia* and imipenem. Overview of all mutations found in each PA01:rfp clone isolated from independent monoculture and *S. maltophilia* co-culture populations evolved in 0, 1, 4 or 8 μg/ml imipenem for 24 days. In intergenic regions, the annotation shows the position of the mutation in nucleotide (nt) distance between genes '(+ first gene / - second gene)', while frameshift annotations display the nt position of the mutation within the affected gene '(mutation position / gene length)'. For amino acid (AA) substitutions and stop codon insertions (*), annotation shows the 'original AA – AA position – new AA' and the codon nt change '(original codon -> new codon)'. Red nucleotides highlight the difference between mutated codons causing a change in AA (blue) or synonymous change (green).
